# Highlighter: An optogenetic system for high-resolution gene expression control in plants

**DOI:** 10.1371/journal.pbio.3002303

**Published:** 2023-09-21

**Authors:** Bo Larsen, Roberto Hofmann, Ines S. Camacho, Richard W. Clarke, J Clark Lagarias, Alex R. Jones, Alexander M. Jones

**Affiliations:** 1 Sainsbury Laboratory, University of Cambridge, Cambridge, United Kingdom; 2 Biometrology, Chemical and Biological Sciences Department, National Physical Laboratory, Teddington, United Kingdom; 3 Department of Molecular and Cellular Biology, University of California, Davis, California, United States of America; University of California San Diego, UNITED STATES

## Abstract

Optogenetic actuators have revolutionized the resolution at which biological processes can be controlled. In plants, deployment of optogenetics is challenging due to the need for these light-responsive systems to function in the context of horticultural light environments. Furthermore, many available optogenetic actuators are based on plant photoreceptors that might crosstalk with endogenous signaling processes, while others depend on exogenously supplied cofactors. To overcome such challenges, we have developed Highlighter, a synthetic, light-gated gene expression system tailored for *in planta* function. Highlighter is based on the photoswitchable CcaS-CcaR system from cyanobacteria and is repurposed for plants as a fully genetically encoded system. Analysis of a re-engineered CcaS in *Escherichia coli* demonstrated green/red photoswitching with phytochromobilin, a chromophore endogenous to plants, but also revealed a blue light response likely derived from a flavin-binding LOV-like domain. We deployed Highlighter in transiently transformed *Nicotiana benthamiana* for optogenetic control of fluorescent protein expression. Using light to guide differential fluorescent protein expression in nuclei of neighboring cells, we demonstrate unprecedented spatiotemporal control of target gene expression. We implemented the system to demonstrate optogenetic control over plant immunity and pigment production through modulation of the spectral composition of broadband visible (white) light. Highlighter is a step forward for optogenetics in plants and a technology for high-resolution gene induction that will advance fundamental plant biology and provide new opportunities for crop improvement.

## Abstract 2

Emerging high-resolution analytic technologies like single-cell sequencing and FRET biosensors for metabolites are revolutionizing the scale of biological hypotheses. This study describes Highlighter, an optogenetic tool for fine-scale eukaryotic gene expression control, and deploys it in plants to enable cell-level hypothesis testing.

## Introduction

Recent development of innovative and enabling high-resolution technologies has furthered the study of cellular processes, metabolic pathways, and regulatory systems. New measurements available to biologists, from single-cell gene expression levels [1,2] to quantification of metabolites in tissues and in individual living cells [3–5], shines new light on the spatial and temporal relationships between quantified analyte and biological phenomena. However, to transcend the limits of correlative studies and establish causation, we must also be able to perturb biological systems with cellular resolution.

Current tools for spatiotemporal perturbation, such as chemically inducible or tissue-specific gene expression systems, can lack the desired resolution and may suffer from a series of additional limitations. For example, chemically inducible systems provide an element of temporal control, but typically depend on inducer molecules to diffuse into organs, tissues, and cells, limiting spatial and temporal resolution of application and removal. Further, they can be expensive and invasive to biological processes due to pharmacological activity and toxicity [6–9]. Correspondingly, cell-type or tissue-specific gene expression tools provide some degree of spatial control but are limited to previously characterized promoters and often lack specificity. However, optogenetic actuators, such as light inducible gene regulatory systems, could provide sought-after high-resolution spatiotemporal control because light can be delivered with exquisite precision and with low toxicity.

One of the first reported synthetic light-controlled gene regulatory systems exploited a light-controlled protein–protein interaction between photoactive plant phytochromes and phytochrome interaction factors to drive reversible association of the split GAL4 transcription factor in *Saccharomyces cerevisiae* [10]. Following this breakthrough, the number of optogenetic actuator systems expanded rapidly from applications of light-controlled ion channels in neuroscience to numerous light-controlled biological processes in many cell types and even subcellular domains in living organisms [11]. Unfortunately, the implementation of optogenetic actuators in plants has proven challenging because plants require light-dark cycling for healthy growth and development. Most available optogenetic systems would be unable to maintain a single activation state under such conditions and thus applications are limited to those that can tolerate corresponding activation-inactivation cycles [11,12]. Furthermore, many optogenetic tools are based on light-responsive proteins from plants, such as PHYB, CRY2, PHOT&ZTL (LOV domains), and UVR8 [13], and may therefore crosstalk with endogenous light signaling pathways, potentially resulting in off-target modulation, or interference with the function of the synthetic optogenetic actuator itself. To minimize such problems, it is routine to orthogonalize system components (i.e., engineer system components to avoid interactions with endogenous components) through mutation or truncation, as exemplified by the orthogonalized PhyB-PIF6 system [8]. Hence, ideal optogenetic actuators for plants will (1) be systems that specifically respond to artificial light stimuli; (2) assume a single activation state under standard plant growth conditions, i.e., light-dark cycling; (3) function as an optically controlled switch with distinct on- and off-states; (4) be orthogonal to plant signaling processes; and (5) not require an exogenously supplied chromophore (see below).

In recent years, major advances have been made towards deploying optogenetic actuators to modulate gene expression in plants. Initially, an infrared-controlled actuator (infrared laser-evoked gene operator–IR-LEGO) [14] was deployed in plants to control gene expression from heat shock promoters with high resolution. However, the use of heat shock could lead to off-target gene induction in the targeted cells. Subsequently, a red-light-controlled actuator, based on the N-terminal domains of PhyB and PIF6 from *Arabidopsis thaliana*, was demonstrated to achieve a high dynamic range of gene expression induction in *Nicotiana tabacum*- and *Physcomitrium patens*-derived protoplasts in response to 660 nm red light. However, 740 nm far-red-light supplementation was needed to repress system activity under white light growth—conditions that affect endogenous phytochrome activity [8]. To minimize potential effects on endogenous light signaling processes, the bacterial green- and yellow-responsive CarH photoreceptor was developed as an optogenetic gene expression switch that responds to wavelengths of light that are minimally absorbed by plants [15]. This orthogonal optogenetic system was deployed in *Arabidopsis* protoplasts showing high induction and low background activity, but the photoactuator system is obligatorily dependent on the vitamin B_12_ derivative, 5′-deoxyadenosylcobalamin (AdoB12), an exogenously supplied photolysis-sensitive chromophore. In a recent advance to address challenges associated with activation control during light-dark cycling, the red-activated PhyB-PIF6 system was combined with an engineered blue-off module, based on the LOV-based transcription factor EL222, to generate a fully genetically encoded optogenetic gene expression system called PULSE. PULSE can be activated with red light when blue light is absent and remains off during light-dark cycling [16]. PULSE represents a major milestone for optogenetics in plants, as demonstrated by its deployment to reversibly control induction of firefly luciferase expression in stably transformed *Arabidopsis*.

Aiming to make an optogenetic gene expression system for plants that is orthogonal, fully genetically encoded and independent of exogenously supplied chromophores, we chose to base our design on the CcaS-CcaR system, a green/red photoswitching transcription control system of cyanobacterial origin [17,18]. CcaS is a light-responsive histidine kinase that phosphorylates the response regulator CcaR, which then initiates transcription from a target promoter with cognate *cis*-regulatory elements (CREs) [17,18]. The CcaS-CcaR system was previously repurposed into synthetic optogenetic gene expression systems for prokaryotic hosts such as *Escherichia coli* [19–24], *Bacillus subtilis* [25], and cyanobacteria [26–28]. Target genes were placed under control of promoters with CcaR CREs and biosynthetic genes for the native chromophore of CcaS, phycocyanobilin (PCB), were exogenously expressed in hosts not naturally producing this chromophore. When repurposing this system for deployment in plants, we hypothesized that CcaS, having homology to plant phytochromes, might accept the endogenously produced phytochromobilin (PΦB) chromophore, which supports photoswitching in plant phytochromes [29]. It was expected that PΦB substitution for PCB in the green/red cyanobacteriochrome CcaS would generate a functional analog and hence circumvent the need for exogenously supplied chromophores. Moreover, the light environment used to sustain robust plant growth might be suitably adjusted to maintain the CcaS system in the same activity state in both the light and dark phases of diurnal growth. Light regimes artificially enriched in activating light could then be used to control this system with potentially minimal perturbation of endogenous signaling processes or photosynthesis itself. By repurposing a system of prokaryotic origin, we potentially also minimize crosstalk between the optogenetic actuator and endogenous plant signaling pathways.

In this work, we describe the design, engineering, and validation of Highlighter, an optogenetic actuator tailored for regulating target gene expression levels in plants with cellular resolution. We engineered Highlighter for function in eukaryotic cells and to efficiently photoswitch with PΦB by mutating the chromophore-binding domain in CcaS with the aim to enable use in plants that naturally synthesize PΦB. We found that target gene expression levels can be specifically repressed with blue light and blue-enriched white light and is active with other light regimes, e.g., green-enriched white light. We also show that this blue-off behavior potentially results from blue light sensing by a CcaS flavin-binding domain distinct from its bilin-binding domain. In *Nicotiana benthamiana* leaves transiently expressing Highlighter, we demonstrated robust optical control over fluorescent protein expression levels, pigment production, and induction of immune responses. We furthermore demonstrate the exquisite spatiotemporal control afforded by optogenetic actuators by using Highlighter to drive contrasting expression states in neighboring cells. Because target gene expression can be modulated by altering the spectral properties of white light, Highlighter’s behavior presents a solution for achieving potentially minimally invasive regulation of target gene expression levels under standard horticultural light regimes without the need to combine systems with opposing properties. Highlighter therefore provides new opportunities for optogenetic perturbation of biological processes with high spatiotemporal resolution in plants.

## Results

**Fig 1:**
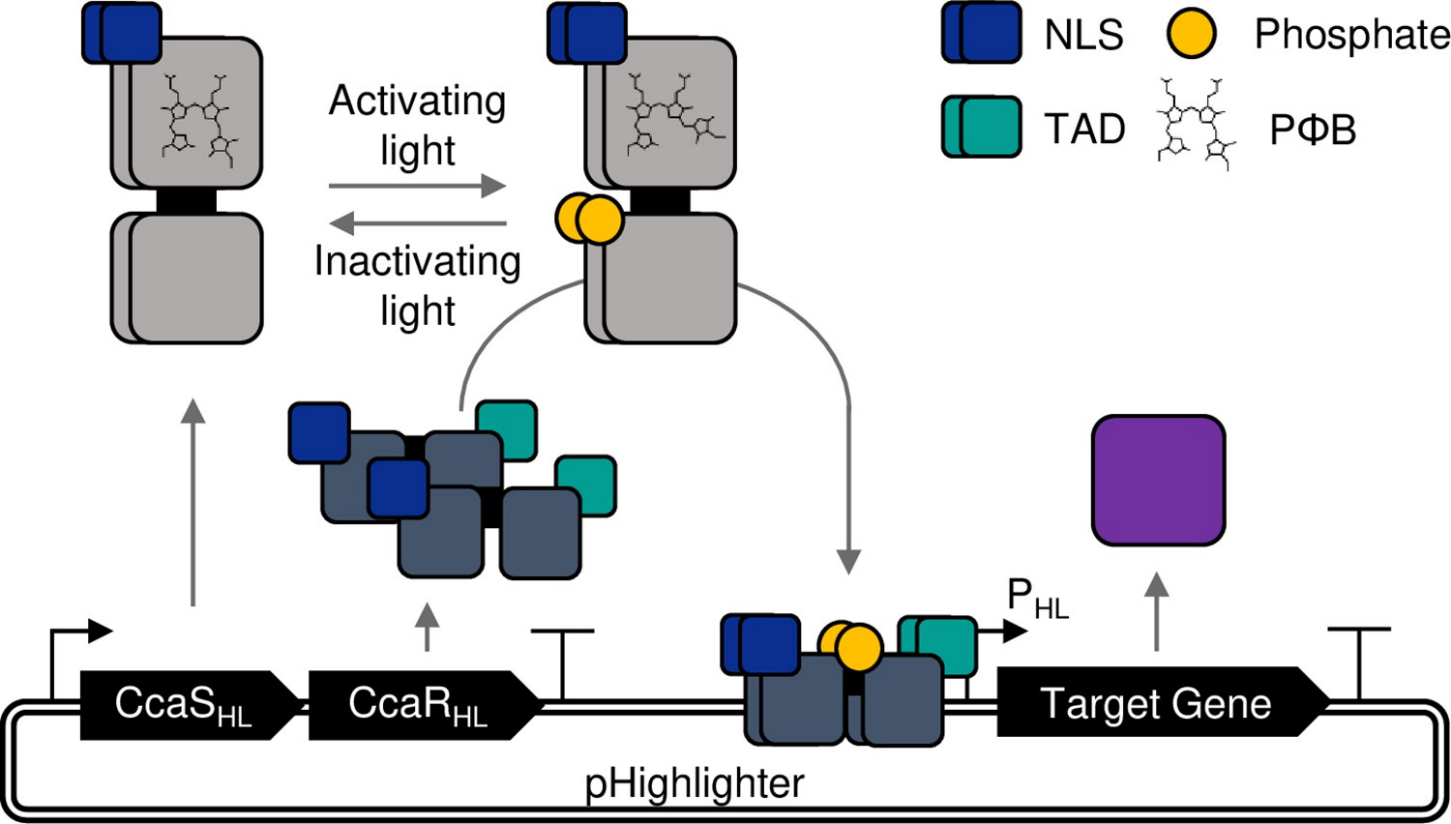
Schematic representation of the Highlighter system and function. Highlighter is the CcaS-CcaR system repurposed for *in planta* function. The repurposed CcaS, CcaR and synthetic promoter are denoted with subscript “HL.” Upon exposure to activating light conditions, CcaS_HL_ phosphorylates CcaR_HL_, which triggers enhanced binding to its cognate promoter, P_HL_, to induce expression of a target gene of interest. CcaS_HL_ and CcaR_HL_ are expressed as a single transcriptional unit from a promoter-terminator expression cassette through use of a F2A_30_ ribosomal skipping sequence. NLS, nuclear localization signal; TAD, transcription activation domain.

The primary challenge of developing Highlighter was to repurpose the CcaS-CcaR system for target gene control in plants, while utilizing the endogenously produced PΦB chromophore. We envisioned that if PΦB supports photoswitching in CcaS, then efficient target gene control *in planta* could be achieved by targeting CcaS-CcaR to the plant nucleus with nuclear localization signals (NLSs), codon-optimizing the system for plant expression, adding eukaryotic trans-activation domains (TADs) to CcaR and engineering a cognate synthetic promoter that incorporates recognition sequences for CcaR adjacent to a minimal plant promoter. Light-activation of the re-engineered CcaS-Highlighter (CcaS_HL_) would, in principle, activate the optimized CcaR-Highlighter (CcaR_HL_), which would bind to the synthetic Highlighter promoter (P_HL_) and recruit the eukaryotic transcriptional machinery—resulting in target gene expression (Fig 1).

### Characterizing chromophore compatibility of the CcaS-CcaR system with PɸB

We first set out to confirm if CcaS can photoswitch effectively with the endogenously produced plant chromophore PΦB. PΦB and the native PCB chromophore of CcaS are structurally similar heme-derived linear tetrapyrroles, which differ by exchange of the 18-ethyl group of PCB with an 18-vinyl group in PΦB [30]. Plant phytochromes PhyA and PhyB, naturally utilizing PΦB, have previously been demonstrated to be able to photoswitch with both chromophores [31–33]. To test the chromophore dependency of CcaS, we used a CcaS-CcaR system variant repurposed for *E*. *coli*, where CcaS and CcaR are expressed together with 2 cyanobacterial enzymes, HO1 (heme oxygenase) and PcyA (ferredoxin-dependent bilin reductase), to synthesize PCB from heme [34,19,20]. To report system activity, superfolder green fluorescent protein (sfGFP) is under the control of an optimized CcaR promoter, P_cpcG2-172_ [20]. Side-by-side comparison of system activity with PCB and PΦB was achieved by substituting *pcyA* with *mHY2*, which encodes a PΦB synthase from *Arabidopsis* lacking its native transit peptide [33].

**Fig 2:**
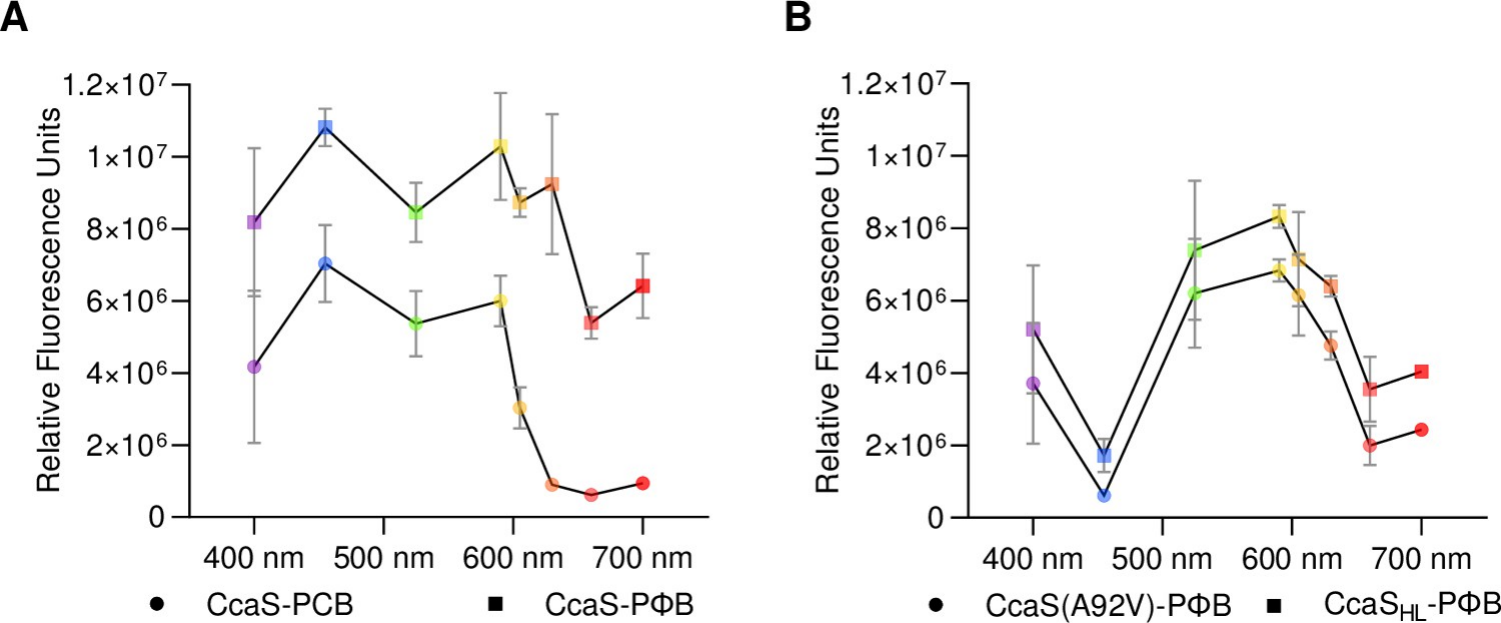
Chromatic response of CcaS-CcaR system variants in *E*. *coli*. (A) Chromophore-dependent photoswitching behavior of the CcaS-CcaR system in *E*. *coli* with PCB and PΦB with unmodified CcaS and CcaR proteins. System output in response to light stimuli was quantified via sfGFP fluorescence and presented as RFUs; RFUs are defined as the mean estimated sfGFP fluorescence from cell cultures with OD600 nm = 0.2. Bacterial cultures were exposed to light stimuli (approximately 10 μmol m^-2^ s^-1^) generated using LEDs with peak wavelength emissions around 400 nm, 455 nm, 525 nm, 590 nm, 605 nm, 630 nm, 660 nm, and 695 nm. (B) System responses for CcaS(A92V) and CcaS_HL_ co-produced with PΦB. Symbols are colored according to light treatment and depict mean fluorescence from 3 replicate experiments, each comprising 3 biological replicates for each light treatment. SEM are presented for each light treatment. The underlying data for panels A and B is in S1 Data. LED, light-emitting diode; PCB, phycocyanobilin; RFU, relative fluorescence unit; sfGFP, superfolder green fluorescent protein.

Expressing the CcaS-CcaR reporter system in PCB-producing *E*. *coIi* cultures yielded a green/red switching transcription actuator system, as expected, which operationally can be activated by wavelengths in the visible spectrum shorter than 630 nm and be repressed by wavelengths greater than 630 nm (Fig 2A). By contrast, in PΦB-producing *E*. *coIi* cultures, sfGFP expression was not robustly light regulated, suggesting that CcaS poorly binds PΦB or does not photoswitch as well as the PCB adduct in *E*. *coli*.

### Improving photoswitching of CcaS with PΦB

To functionally tune CcaS for efficient photoswitching with PΦB, we selected residues for mutagenesis in and around the chromophore binding pocket of CcaS: specifically, conserved amino acid residues within the chromophore-binding cGMP phosphodiesterase/adenylyl cyclase/FhlA (GAF) domain of CcaS homologs that utilize PΦB. Sequence alignments for cyanobacteriochromes (NpR6012g4, TePixJ, FdRcaE, SyCcaS, and SyCph1), plant phytochromes (AtPhyA and AtPhyB) and bacteriophytochromes (PsBphP and DrBphP) revealed candidate amino acids potentially associated with PΦB utilization (S1A and S1B Fig). The A92V mutation in the CcaS-GAF domain dramatically improved photoswitchable transcriptional regulation with PΦB in *E*.*coli* while other tested mutations did not (Figs 2B and S1C). CcaS(A92V) with PΦB exhibited green/red regulation, similar to wild-type CcaS with PCB, but the former appears to require slightly longer wavelengths of light, i.e., red and far-red light from 660 nm, to switch off sfGFP expression. A more profound difference in spectral response was the blue-off behavior of CcaS(A92V) with PΦB, not seen for the unmodified CcaS with PCB. Blue light treatment with wavelengths around 455 nm efficiently lowered system output levels, as reported via the diminished sfGFP fluorescence.

### Modifying CcaS for function in plants

The next step towards repurposing the CcaS-CcaR system for efficient transcription control in plants was to target CcaS(A92V) and CcaR to the plant nucleus by fusing NLS domains to both proteins. For CcaS, this entailed removal of the N-terminal transmembrane domain (TMD) to release the photoreceptor from the cell membrane. We tested in *E*. *coli* whether replacing the TMD with an NLS produced a viable CcaS(A92V) before continuing to modify the system for deployment *in planta*. We found that CcaS(A92V) with the NLS substitution, hereafter CcaS_HL_, with PΦB had a slightly reduced dynamic range of response, compared to CcaS(A92V) with PΦB, but that the modification did not appear to further change the photoreceptor’s photoswitching properties (Fig 2B).

To investigate the response of CcaS_HL_ with PΦB to different illumination wavelengths, we heterologously expressed and purified a hexahistidine-tagged CcaS_HL_ holo-protein from *E*. *coli*. Spectroscopic data confirm that the recombinant PΦB adduct of CcaS_HL_ is reversibly photoswitched between red-absorbing (active) and green-absorbing (inactive) states (S2 Fig), similar to PCB adducts of the CcaS-GAF domain and truncated CcaS (CcaS without the TMD) expressed in *E*. *coli* or in *Synechocystis* sp. PCC 6803 [17]. In comparison with these PCB adducts, photoconversion of the recombinant PΦB adduct of CcaS_HL_ between each state appears less complete. For simplicity, we will continue to refer to them as the red- and green-absorbing states, but they can be more fully described as “red/green-absorbing” and “predominantly green-absorbing,” respectively, an important distinction to some of the discussion below. An isosbestic point exists at approximately 605 nm between these states, consistent with the transition between the active and inactive optogenetic behavior we observe in *E*. *coli* (Fig 2B). Blue light illumination has the same effect on the absorption spectrum as green light (S2C and S2E Fig), i.e., it converts the green-absorbing state to the red-absorbing state. This suggests blue light should switch CcaS_HL_ into the active state, which appears to contradict the observed effect of blue light in *E*. *coli* (Fig 2B), where it switches off sfGFP expression.

Could this effect of blue light be explained by another difference observed between the spectra of the PΦB adduct of CcaS_HL_ and the PCB adduct of the CcaS GAF domain [17]? We noted additional, unexpected absorption peaks around 475 nm and 445 nm, which resemble signal from oxidized flavin partially obscured by the UVA absorption peaks from PΦB. Together, these 2 peaks in the blue have the sort of defined vibrational structure observed when oxidized flavin is protein bound (e.g., LOV2 from phototropin, [35]). Moreover, the magnitude of the UVA peak we observe for the green-absorbing state of the PΦB chromophore in CcaS_HL_ is greater relative to the green peak than one might expect [17], consistent with additional absorption in this region from oxidized flavin. It is therefore possible that oxidized flavin, e.g., flavin mononucleotide (FMN), is bound to the PAS (Per-ARNT-Sim) domain of CcaS_HL_, similar to LOV domains. To investigate this further, we acquired the fluorescence emission spectrum (S3 Fig), exciting the red-absorbing state (from which blue light causes no further photoisomerization of the PΦB, S2F Fig) near the peak of the putative flavin absorption signal (445 nm). Because fluorescence signals typically come from the lowest-lying excited state, emission from flavin would not be obscured by transitions associated with higher-lying states of the PΦB. This is evident in S3 Fig, where a broad emission with peaks at 495 nm and 515 nm is, like the visible absorption signals, strongly reminiscent of the equivalent FMN spectrum from the phototropin LOV2 domain [35]. The peaks at 628 nm and 666 nm are likely from transitions associated with the residual broad green absorption in the red-absorbing state of the PΦB chromophore in the GAF domain of CcaS_HL_. Interestingly, the second PAS domain of CcaS exhibits high sequence similarity to LOV2 domains [17], for example, 39.8% identity to the LOV2 domain of *Arabidopsis* PHOTOTROPIN 1. The PAS domain in CcaS has several of the key flavin-coordinating residues observed in flavin-binding LOV domains, e.g., the CcaS G_433_KTPRVLQ_440_ motif, excepting the conserved cysteine that is involved in photochemical flavin-adduct generation in LOV domains. It is possible that the counterintuitive effect of blue light on CcaS_HL_ in *E*. *coli* (Fig 2B) is owing to a noncanonical effect on flavin bound to this cysteine-less, LOV-like domain, as previously shown for several other LOV receptors [36].

### Deploying Highlighter in transiently transformed *N*. *benthamiana* for optogenetic control of target gene expression

Having engineered CcaS_HL_ for nuclear targeting and for photoswitching with PΦB, we optimized the CcaS-CcaR system for plant deployment. First, to regulate target gene expression levels *in planta*, we needed to enable CcaR to effectively recruit the plant transcriptional machinery and initiate transcription, and second, we needed to create a plant compatible promoter recognized by CcaR. To achieve this, we converted CcaR into a eukaryotic transcription factor by adding a synthetic, C-terminal VP64 transcription activation domain [37]. For efficient activation by nuclear targeted CcaS_HL_, we included an N-terminal NLS domain (the resulting nlsCcaR:VP64 is hereafter referred to as CcaR_HL_). In parallel, we designed a synthetic cognate promoter for CcaR_HL_, named P_HL_, by placing 3 CcaR binding motifs 5′ to a 35S minimal promoter [38]. The binding motifs were spaced evenly around the DNA helix, offset relative to one another at approximately 120° angles, to maximize the chance of having at least one P_HL_-bound CcaR_HL_ being advantageously oriented to the 35S minimal promoter. Finally, considering that both CcaS and CcaR are of prokaryotic origin, we codon-optimized CcaS_HL_ and CcaR_HL_ for plant expression. Together, these repurposed components comprise the Highlighter system.

To demonstrate optogenetic control of target gene expression with Highlighter *in planta*, we constructed a series of vectors for deployment in transiently transformed *N*. *benthamiana*. Because heterologous gene expression levels can be variable in transient expression systems, we devised a ratiometric fluorescent reporter system for tracking system activity. To report target gene regulation, we placed nlsedAFPt9 [39], a nuclear targeted yellow fluorescence protein (YFP) variant, under P_HL_ control, creating a YFP response module (P_HL_::nlsedAFPt9::T_nos_). For normalization of target gene expression levels to system gene expression levels, we transcriptionally linked expression of a second fluorescent reporter protein, nlsTagRFP, a nuclear targeted red fluorescence protein, with constitutive CcaS_HL_ and CcaR_HL_ expression. Transcriptional linkage of CcaS_HL_, nlsTagRFP, and CcaR_HL_ from a single promoter-terminator cassette was achieved with 2 interposing F2A_30_ ribosomal skipping sequences [40], effectively creating a single Highlighter expression module (P_UBQ10_::CcaS_HL_:F2A_30_:nlsTagRFP:F2A_30_:CcaR_HL_::T_RBCS_). Together, these 2 modules comprise the Highlighter(YFP) system (S1 Table).

**Fig 3:**
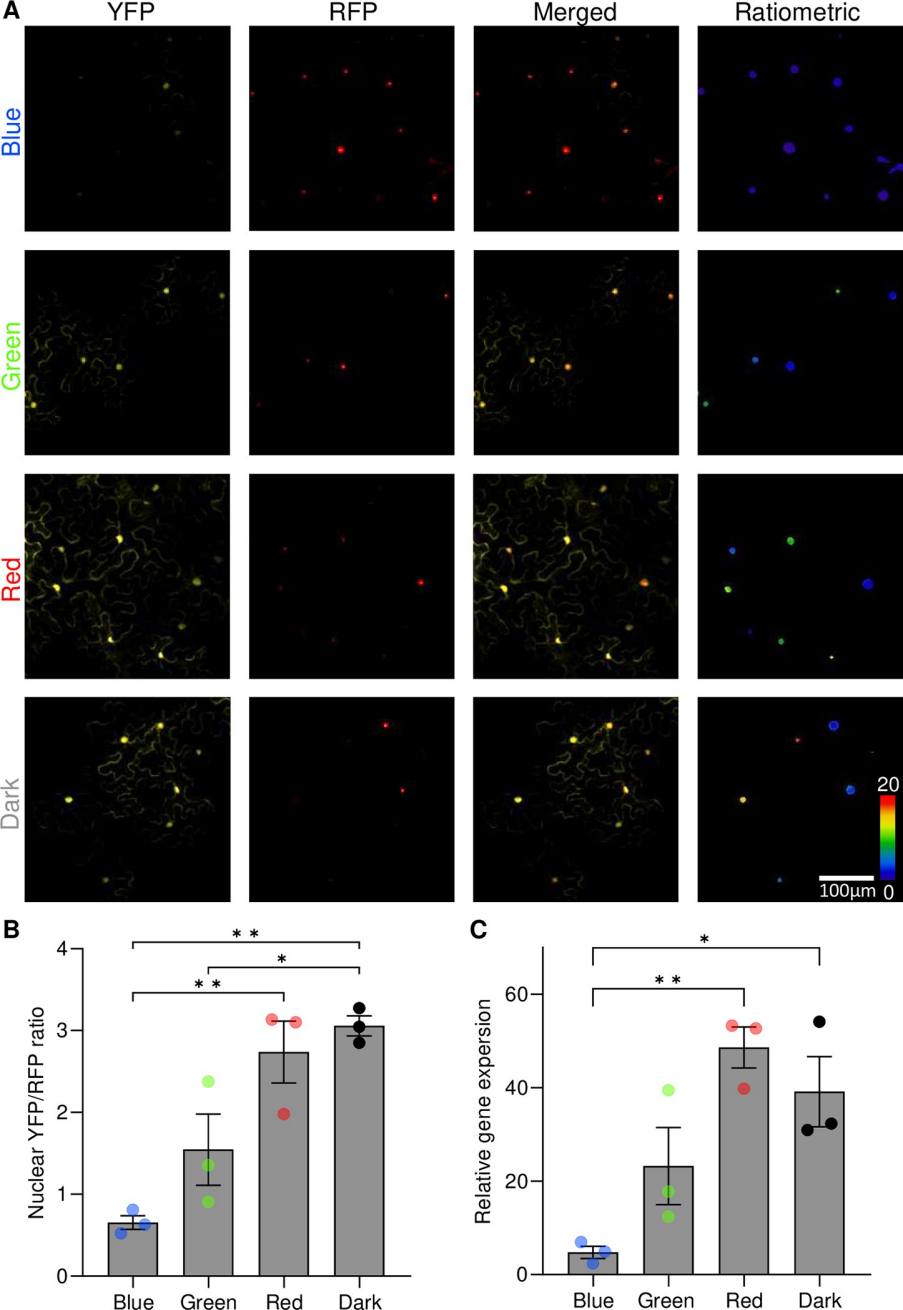
Deployment of Highlighter in *N*. *benthamiana* leaves for controlling fluorescent protein levels with monochromatic light and darkness. *N*. *benthamiana* leaves were infiltrated with *A*. *tumefaciens* for delivery of Highlighter(YFP) and kept in darkness overnight before receiving continuous treatments for approximately 3 days with blue, green, and red light or darkness. Light was delivered with LEDs (100 μmol m^-2^ s^-1^) with peak wavelength emissions λ ~ 455 nm, 525 nm, and 660 nm, respectively. (A) Representative confocal images demonstrating nuclear YFP and RFP fluorescence in light-treated samples, alongside merged images of the YFP and RFP fluorescence and finally the calculated YFP/RFP ratios (ratiometric). (B) Quantification of YFP/RFP ratios in light-treated samples. (C) Relative gene expression levels in light-treated samples. In (B) and (C), means and SEM are presented for 3 biological independent experiments. Individual experimental means are depicted with circles colored according to light treatment. *n* per mean, i.e., per circle, in (B) is 22 to 168 nuclei from 3 to 4 infiltrated spots across 3 to 4 leaves. In (C), for each biological replica mean, leaf material from 4 infiltrated spots across 4 leaves were combined and analyzed using quadruple technical qPCR replicates. Highlighter(YFP) Vector ID: pBL413-024-257 (S1 Table). * *P* < 0.05 and ** *P* < 0.01. Leaves were spot infiltrated with OD600 nm = 0.4 *A*. *tumefaciens* cultures. The underlying data for panels B and C is available in S2 Data. LED, light-emitting diode; YFP, yellow fluorescent protein.

In *N*. *benthamiana* leaves infiltrated with *Agrobacterium tumefaciens* for transient transformation with Highlighter(YFP), we compared the ratio of nuclear YFP/RFP fluorescence under different light conditions. We observed significantly lower YFP/RFP ratios in samples kept in continuous blue light (λ ~ 455 nm), indicating lower relative target gene expression levels, as compared to leaves subjected to continuous green light (λ ~ 525 nm), red light (λ ~ 660 nm), or darkness (Fig 3A and 3B). The lower expression in blue relative to green was expected from results in *E*. *coli*, but elevated Highlighter target gene expression in red light and darkness diverged from *E*. *coli* behavior. While this indicates that Highlighter likely functions differently in *N*. *benthamiana* leaves, the system remained responsive to color changes in the visible spectrum, particularly blue-off. Target gene expression levels were additionally verified to correlate with the observed fluorescence ratios by qRT-PCR (Fig 3C). The observed pattern of light responsiveness of Highlighter(YFP) was not observed for a positive control construct in which P_HL_ was exchanged with a 35S promoter variant to constitutively express YFP nor was it observed for negative control constructs lacking either CcaS_HL_ or CcaR_HL_ from the Highlighter expression module (ΔCcaS_HL_, ΔCcaR_HL_, S4 Fig). Maximum target gene expression levels for Highlighter(YFP), i.e., the YFP/RFP ratio, in red light or darkness was lower than for constitutive YFP expression and the minimum target gene expression level for Highlighter(YFP) in blue light was greater than for Highlighter(YFP) ΔCcaS_HL_ and ΔCcaR_HL_. Taken together, the results indicate that Highlighter is a functional light-controlled gene expression switch in plants, but with some leaky expression in blue light and submaximal induction in activating conditions (S4 Fig).

**Fig 4:**
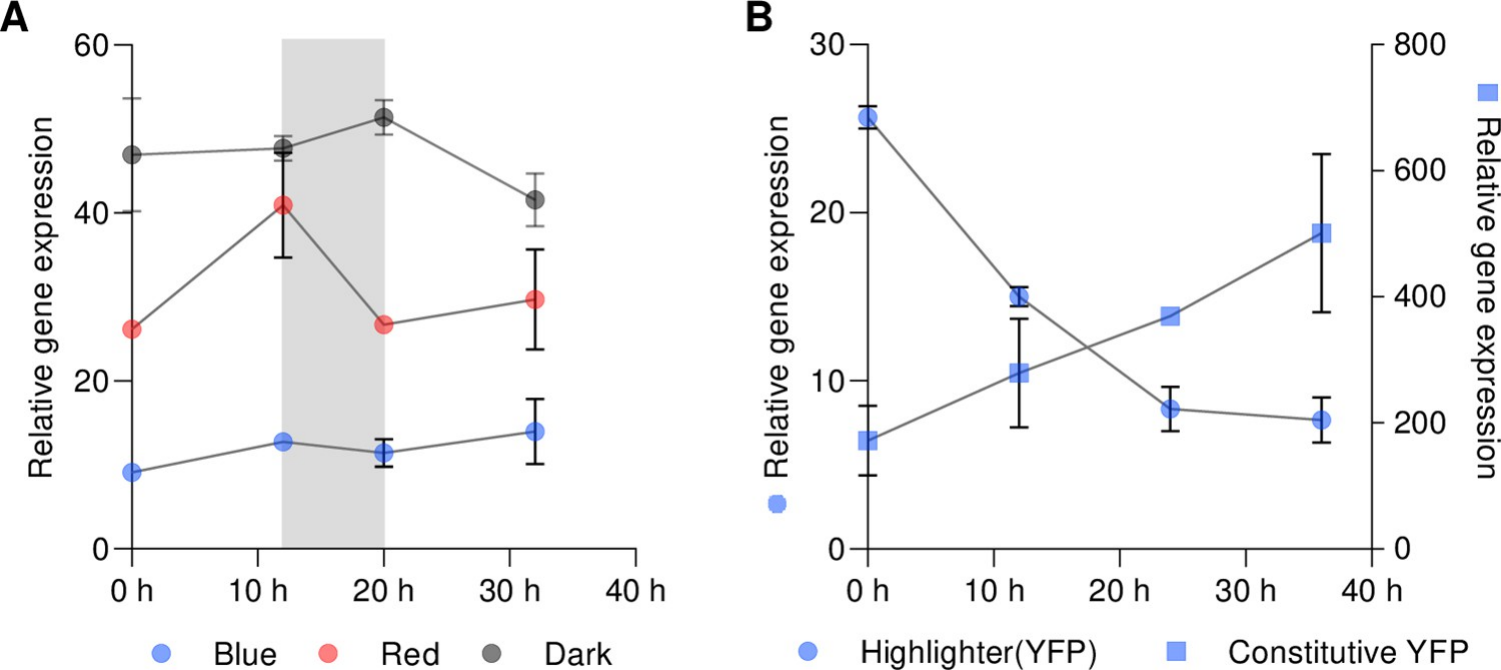
Transcriptional behavior of the Highlighter system in *N*. *benthamiana* leaves during light-dark cycling and system reversibility. (A) Quantification of YFP target gene transcript levels during light-dark cycling in *N*. *benthamiana* leaves transiently transformed with Highlighter(YFP). Prior to subjecting infiltrated leaves to light-dark cycling, the leaves were kept dark overnight and pretreated for 2 days with monochromatic blue light, red light, or kept in darkness. The samples then continued their previous light treatments for 12 h, before being dark treated for 8 h, and were returned to their original light treatments for another 12 h. The gray column in the graph denotes the dark treatment. (B) Highlighter system reversibility test. *N*. *benthamiana* leaves infiltrated with Highlighter(YFP) or the Highlighter control construct for constitutive YFP expression were kept dark overnight and red light treated for 2 days with monochromatic red light before (at 0 h) being subjected to monochromatic blue light. Means and SEM are presented for 3 biological independent experiments for each time point. For each treatment and time point, in each of the biological replicates, leaf material from 4 infiltrated spots across 4 leaves were combined for analysis using quadruple technical qPCR replicates. Highlighter(YFP) Vector ID: pBL413-024-257; Constitutive YFP Vector ID: pBL413-024-259 (S1 Table). Leaves were spot infiltrated with OD600 nm = 0.4 *A*. *tumefaciens* cultures. The underlying data for panels A and B is in S3 Data. YFP, yellow fluorescent protein.

Standard horticultural light environments include light-dark cycling. To evaluate the transcriptional effects of light-dark cycling, we included an 8 h dark period within monochromatic blue light, red light and dark treatments of *N*. *benthamiana* leaves transiently transformed with Highlighter(YFP). Time series qRT-PCR analysis revealed that continuous darkness and red-dark-red cycling resulted in consistently high target gene expression, as expected (Fig 4A). Interestingly, the blue light treatment interrupted with an 8 h dark treatment maintained lower target gene expression at all time points (Fig 4A). This indicates that 8 h of darkness, in contrast to constitutive darkness, is not sufficient to activate Highlighter target gene induction.

To determine whether Highlighter system activity could be reversed, we investigated the temporal dynamics of a switch from red light to inactivating blue light (Fig 4B). Time series qRT-PCR analysis revealed that following red light, blue light treatment lowered target gene expression over a period of 24 h while constitutive YFP expression from the control construct increased throughout the experiment (Fig 4B). These results indicate that transcript levels require an extended time period to decrease, possibly resulting from incomplete or slow blue light inactivation of CcaS_HL_ or slow deactivation of CcaR_HL_, but nonetheless confirm that Highlighter target gene activation can be reversed.

### Optogenetic control of target gene expression with cellular resolution

**Fig 5:**
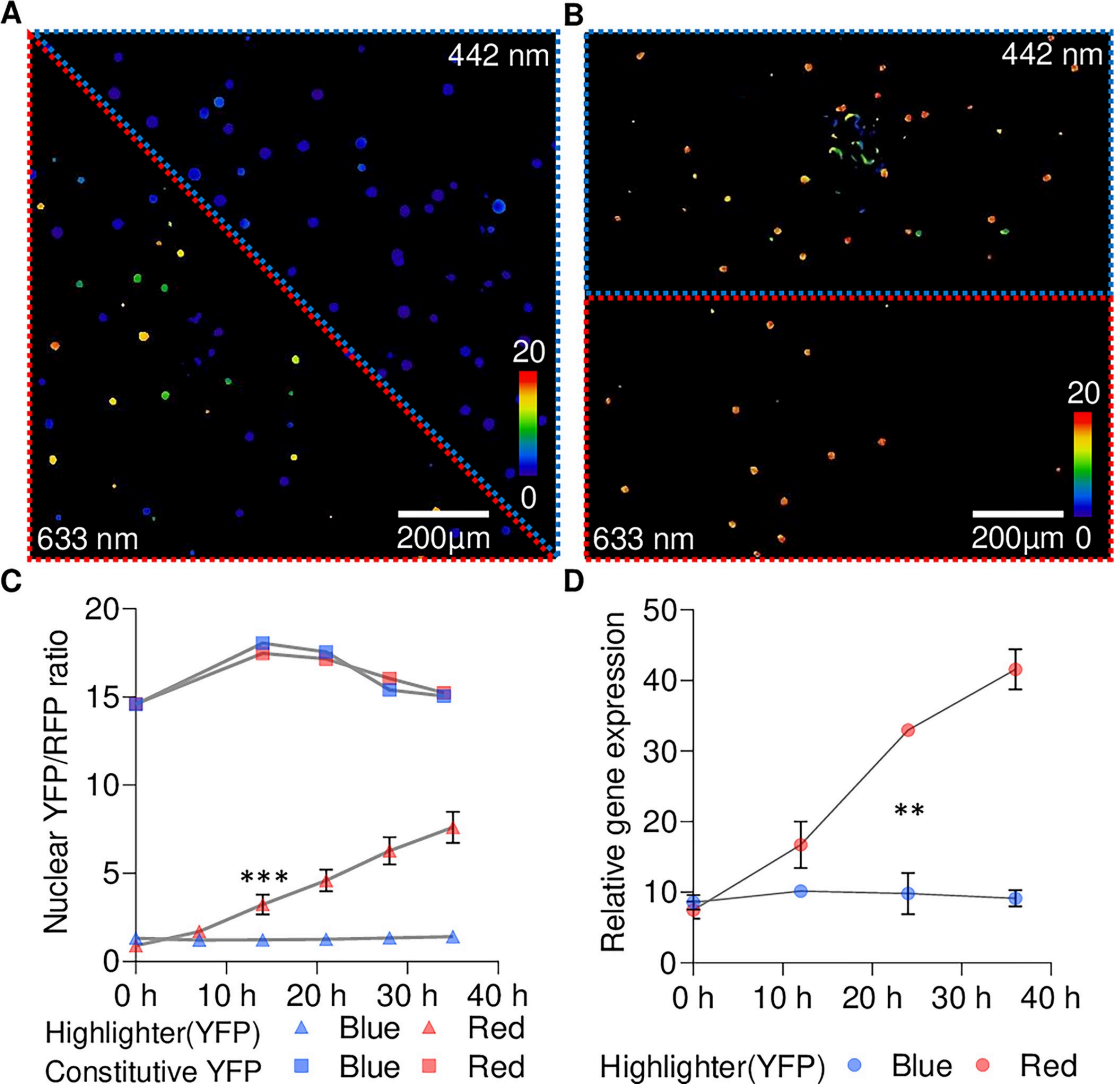
High-resolution control of target gene induction in *N*. *benthamiana* leaves using laser illumination. After infiltration with Agrobacterium for delivery of Highlighter(YFP) (A) or the constitutive YFP expression control (B), samples were kept in the dark overnight prior to continuous blue light treatment with LEDs (100 μmol m^-2^ s^-1^). Samples were blue light treated until 2.5 days post infiltration to minimize target gene expression and then subjected to blue and red light treatments with lasers (initiation of laser treatments are defined as 0 h in (C)); 442 nm blue and 633 nm red lasers were used to irradiate the area outlined in blue and red, respectively. Images in (A) and (B) are ratiometric representations of the YFP/RFP ratios observed after 38 h of light treatment (five 7 h light treatments interrupted by confocal imaging). Images are sum projections of z-stacks spanning multiple cell layers. Cellular resolution measurements of individual nuclear YFP/RFP ratios for Highlighter(YFP) are available in S6 Fig. (C) Temporal quantification of YFP/RFP ratios for laser-based target gene induction in (A) and (B). Highlighter(YFP) data is represented with triangles and data for constitutive YFP expression is represented with squares. Mean and SEMs are presented for each time point. The first time point where there is a significant difference between the nuclear YFP/RFP ratios in the 442 nm and 633 nm treated Highlighter(YFP) infiltrated area is marked with *** (*P* = 0.0003). (D) Temporal quantification of YFP target gene transcript levels during blue or red light treatments. Again, samples were kept in the dark overnight and treated continuously with blue light until 2.5 days post infiltration (here defined as 0 h) to minimize target gene expression levels. Infiltrated leaves were then exposed to blue and red LED light treatments (λ ~ 455 nm and 660 nm, respectively, 100 μmol m^-2^ s^-1^) for 36 h and leaf tissue was sampled every 12 h. Means and SEM are presented in (D) for 3 biological independent experiments. For each time point in each of the biological replicates in (D), leaf material from 4 infiltrated spots across 4 leaves were combined for analyzed using quadruple technical qPCR replicates. The first time point with a significant difference between the relative gene expression levels in blue and red treated Highlighter(YFP) samples is marked with ** (*P* = 0.0015). Highlighter(YFP) Vector ID: pBL413-024-257; Constitutive YFP Vector ID: pBL413-024-259 (S1 Table). Leaves were spot infiltrated with OD600 nm = 0.4 *A*. *tumefaciens* cultures. The underlying data for panels C and D is in S4 Data. LED, light-emitting diode; YFP, yellow fluorescent protein.

Tunable system activity with cellular spatiotemporal resolution is a highly desirable property of optogenetic actuators and is required to answer a multitude of high-resolution biological hypotheses. To explore the spatiotemporal limits for gene induction with Highlighter, we locally irradiated neighboring regions in transiently transformed *N*. *benthamiana* leaf discs with 442 nm and 633 nm lasers using a Fluorescence Recovery After Photobleaching (FRAP) module at sub-photobleaching levels on a confocal microscope. In leaf discs transformed with Highlighter(YFP), the YFP/RFP ratios remained low in the region treated with the 442 nm laser and steadily increased over time in the region treated with the 633 nm laser (Fig 5A and 5C). Transcript levels, assayed by qRT-PCR, also correlated with this trend when exposed to similar red and blue stimuli generated with light-emitting diode (LED) lights (relative Fig 5D and absolute S5A and S5B Fig). To investigate if the lower YFP/RFP ratios observed during the 442 nm laser treatments could be a result of preferential photobleaching of YFP, rather than lower YFP expression, we also tested the constitutive YFP response module variant. We did not observe YFP/RFP ratio photoswitching in leaf discs when YFP was constitutively expressed nor YFP photobleaching by either laser (Figs 5B, 5C, S5C and S5D). Together, these results indicate that, similar to the blue light inactivation (Fig 4B), full system activation following blue light treatment requires an extended period of time, i.e., greater than half a day, possibly owing to slow or no reactivation of CcaS_HL_ after blue light inactivation. This analysis provides first proof-of-concept that Highlighter can be deployed for cell-level expression control *in planta* using light stimuli in the visible spectrum.

### Optogenetic control of plant immunity

Highlighter was developed to assert control over biological processes *in planta*. Having demonstrated that Highlighter could be deployed to regulate target gene expression levels, we aimed to provide proof-of-concept for application by modulating plant immune responses. Specifically, we tested whether Highlighter could be used for optogenetic control of the hypersensitive response (HR), a suite of responses activating effector-triggered immunity in plants [41]. High-resolution optogenetic control of HR in transiently transformed *N*. *benthamiana* would enable future experiments with sufficient spatiotemporal resolution for investigating the mechanisms underlying HR progression during infection.

For this demonstration, we chose to take optogenetic control over effector-triggered immunity with Highlighter by modulating expression levels of an auto-active immune regulatory protein. During effector-triggered immunity, plant cells recognize pathogen effector proteins with intracellular immune receptors called NLRs (nucleotide-binding and leucine-rich repeat). NLRs trigger innate immune responses, including rapid programmed cell death and accumulation of phenolic compounds whose UV-autofluorescence can be detected in the local region surrounding an infection. Many sensor NLRs rely on helper NLRs, such as NLR-REQUIRED FOR CELL DEATH (NRC) proteins, to effectively translate effector recognition into HR [42]. A D478V mutation in NRC4 from *N*. *benthamiana* (NRC4^D478V^) creates an auto-active protein that can activate HR in the absence of infection when transiently overexpressed in *N*. *benthamiana* leaves [42]. NRC4^D478V^ thus presented an excellent target for Highlighter control of plant immunity.

**Fig 6:**
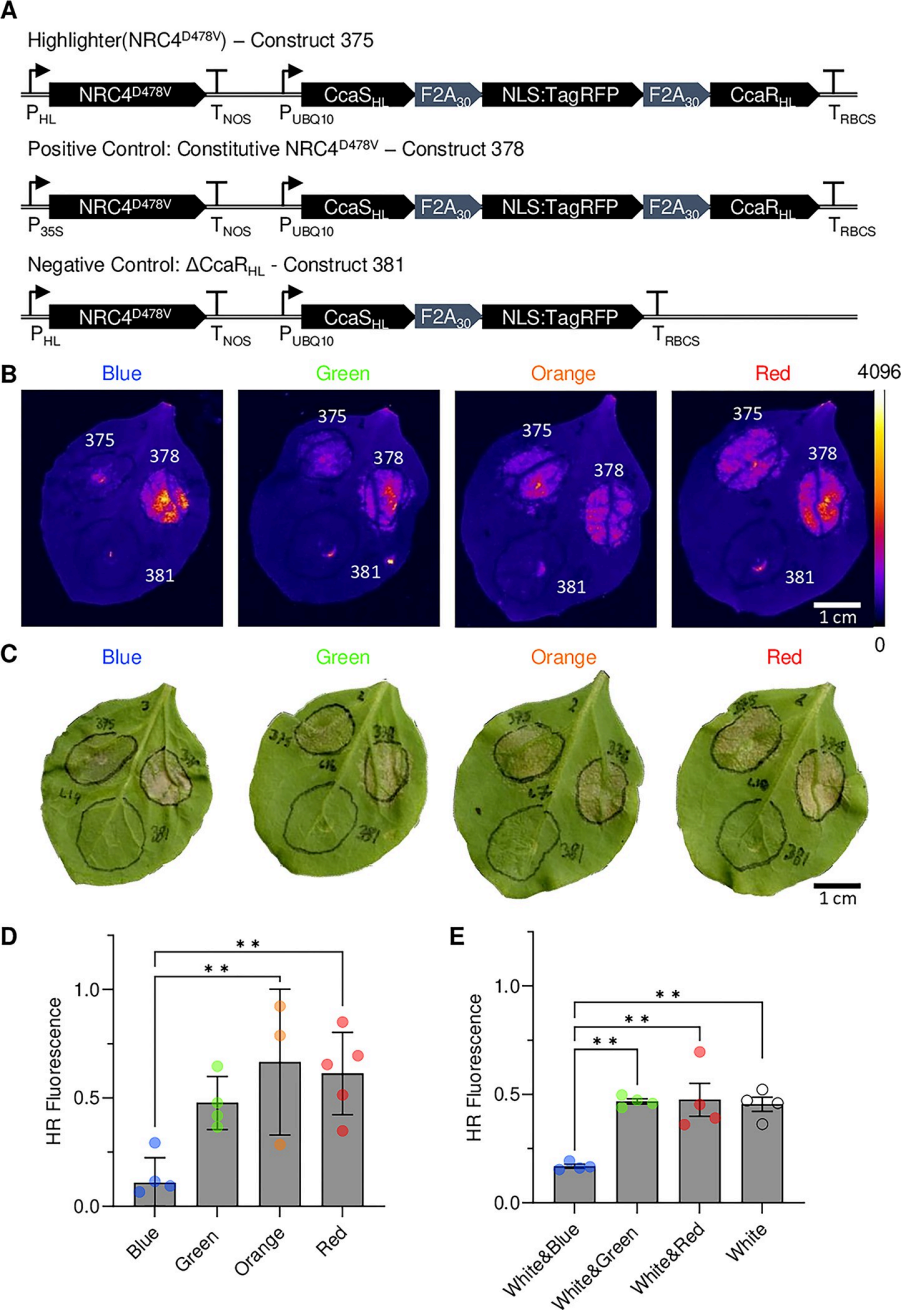
Highlighter controlled immune responses in transiently transformed *N*. *benthamiana* leaves. (A) Highlighter constructs used to assert control over immune responses in *N*. *benthamiana* leaves in response to light treatment. In Highlighter(NRC4^D478V^), construct 375, NRC4^D478V^ is under control of the Highlighter system, via P_HL_, whereas the Highlighter construct for constitutive NRC4^D478V^ expression, construct 378, is a positive control used for normalization. The Highlighter construct missing CcaR_HL_, construct 381, is a negative control construct used for background subtraction. (B) Representative images of HR-induced fluorescence in response to NRC4^D478V^ expression under blue, green, orange, and red light; λ ~ 455 nm, 525 nm, 630 nm, and 660 nm, respectively. LUT is the Fire LUT, ImageJ. Strong UV-fluorescent signals are observed at the center of Agrobacterium-infiltrated spots due to tissue damage from the syringe infiltration and is also clearly observed in (C). (C) Images of leaves in (B) for demonstrating HR-associated cell death progression. Images in panels B and C were acquired approximately 4 DPI. (D) Quantification of HR regulated by Highlighter-controlled NRC4^D478V^ expression under blue, green, orange, and red LED light (λ ~ 455 nm, 525 nm, 630 nm, and 660 nm, 100 μmol m^-2^ s^-1^ light intensity). $HR\;fluorescence=\frac{Inducible\;construct\;fluorescence-Negative\;control\;fluorescence}{Positive\;control\;fluorescence-Negative\;control\;fluorescence}$ (E) Highlighter control of HR levels in white light regimes supplemented with blue, green, or red light. Enriched white light regimes were defined as 50 μmol m^-2^ s^-1^ light from a 5,700 K white light LED channel supplemented with 50 μmol m^-2^ s^-1^ light from blue, green, or red channels with λ ~ 455 nm, 525 nm, and 660 nm, 100 μmol m^-2^ s^-1^ total light intensity. Mean and SEM are presented for each treatment and symbols represent average HR responses from 3 to 5 biological repeats; *n* per biological average is 3 to 12 for monochromatic data in (D) and 5–14 for enriched white light data (E). * *P* < 0.05 and ** *P* < 0.01. Highlighter(NRC4^D478V^) Vector ID: pBL413-037-375; Constitutive NRC4^D478V^ Vector ID: pBL413-037-378; Highlighter(NRC4^D478V^) ΔCcaR_HL_ Vector ID: pBL413-037-381 (S1 Table). Leaves were spot infiltrated with OD600 nm = 0.2 *A*. *tumefaciens* cultures. The underlying data for panels D and E is in S5 Data. DPI, days post infiltration; HR, hypersensitive response; LED, light-emitting diode.

We constructed Highlighter(NRC4^D478V^), as well as positive and negative controls (i.e., Constitutive NRC4^D478V^ and ΔCcaR_HL_, respectively), to regulate and evaluate effects of NRC4^D478V^ expression in *N*. *benthamiana* leaves (Fig 6A). To verify that constitutive expression of NRC4^D478V^ activates HR, we tracked the buildup of fluorescent compounds [43] during *Agrobacterium*-mediated transient expression with the positive control. Strong, localized fluorescence was observed and readily imaged upon UV-A excitation (Fig 6B). The same was not observed for the negative control construct, which lacks the response regulator, CcaR_HL_ (Fig 6B). This suggested that fluorescence buildup could be used as proxy for NRC4^D478V^ expression and HR induction (Fig 6B and 6C). For quantification of HR-associated fluorescence in all experiments, negative control samples were used for background fluorescence subtraction while positive control samples were used for fluorescence normalization. During *Agrobacterium*-mediated transient expression of Highlighter(NRC4^D478V^), we observed markedly lower relative fluorescence in *N*. *benthamiana* leaves kept in continuous blue light (λ ~ 455 nm), compared to green light (λ ~ 525 nm), orange light (λ ~ 630 nm), and red light (λ ~ 660 nm) (Fig 6B and 6D). These results were in clear agreement with our previous results for optogenetic control of YFP/RFP ratios in transiently transformed *N*. *benthamiana* leaves (Fig 3).

In modern horticultural environments, light typically originates from sunlight or broad-spectrum white light comprising LED combinations that support robust plant development. Consequently, to test if gene expression could be controlled under broad-spectrum white light conditions, we compared treatments with white light from LEDs (100 μmol m^-2^ s^-1^) to combinations of white LED light (50 μmol m^-2^ s^-1^) mixed with blue, green, or red LED light (50 μmol m^-2^ s^-1^). These light regimes were designed to maintain a constant total light intensity of 100 μmol m^-2^ s^-1^ across all light treatments. We observed low HR-associated fluorescence in white light modulated with blue light and high HR-associated fluorescence in white light modulated with green or red light (Fig 6E). This confirms that Highlighter is an optogenetic system that can control a biological process in plants grown in mixed light environments.

### Optogenetic control of a chromogenic visual reporter

**Fig 7:**
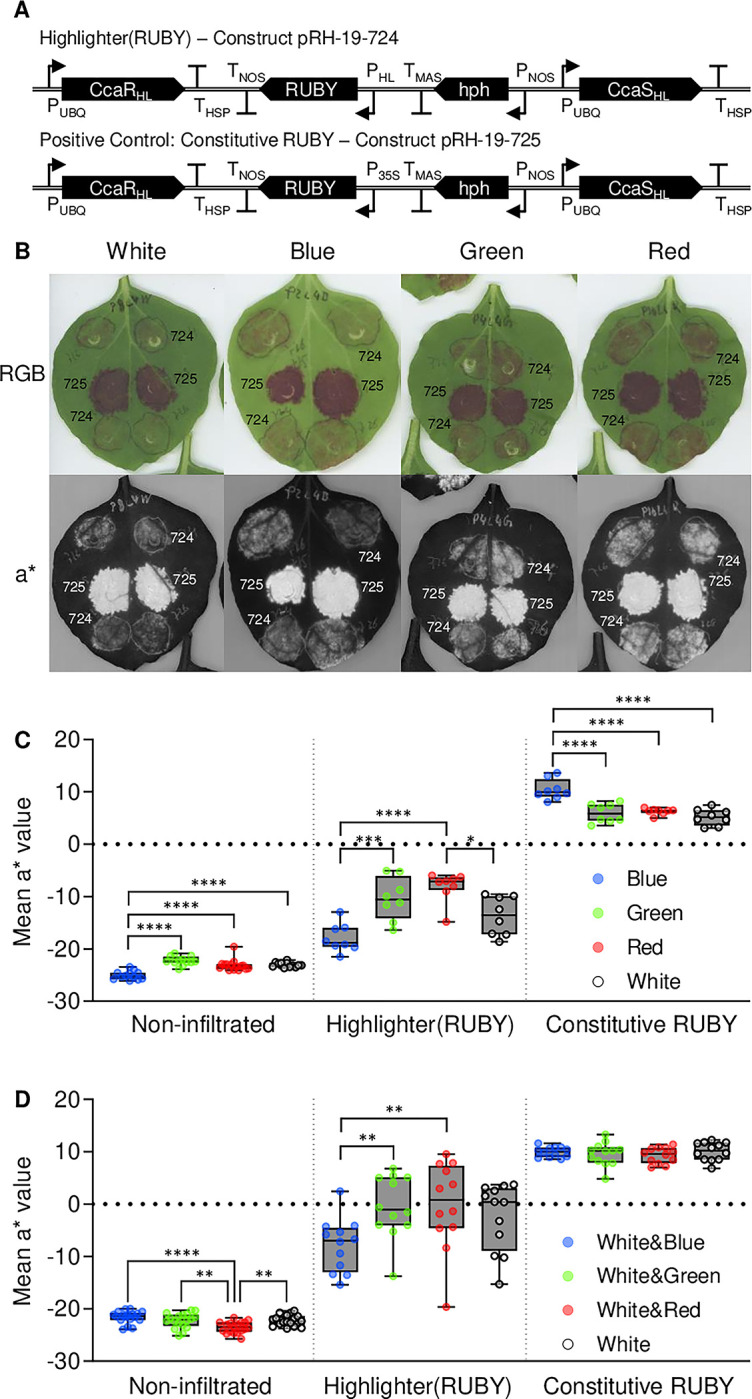
Comparison of Highlighter controlled expression of the betalain-producing RUBY reporter in monochromatic and broad-spectrum white light conditions. *N*. *benthamiana* leaves were infiltrated with the betalain-producing Highlighter(RUBY) reporter construct (Vector ID pRH-19-724 (S1 Table)) and a positive control for constitutive RUBY expression (Vector ID pRH-19-725 (S1 Table)). Plants were moved from darkness to monochromatic light or broad-spectrum white light treatments after 12 h. Monochromatic light treatments were 100 μmol m^-2^ s^-1^ blue (λ ~ 455 nm), green (λ ~ 525 nm), and red (λ ~ 660 nm) light. White light and modulated white treatments were similarly either 100 μmol m^-2^ s^-1^ white light (5,700 K) or mixes of 50 μmol m^-2^ s^-1^ white light with 50 μmol m^-2^ s^-1^ light from the aforementioned blue, green, and red light LEDs. Hence, the light intensities in all applied light regimes were 100 μmol m^-2^ s^-1^. (A) Infiltrated leaves treated with white light or monochromatic blue, green, or red light. Representative images are presented in RGB color space in the top row and their respective a* component, i.e., the red versus green component of the Commission Internationale de l´Eclairage L*a*b* color space (CIELAB), are presented in the row below. (B, C) Quantification of mean a* values for non-infiltrated spots, Highlighter(RUBY) infiltrated spots, and spots infiltrated with a constitutive RUBY expression control. The monochromatic and mixed light experiments were completed 2 and 3 times, respectively, with similar results. Box and whiskers plots, min to max, are presented for each treatment and symbols represent average a* values from infiltrated spots. The white light and monochromatic data in (B) comprises 8 to 15 infiltrated spots per light treatment. The white light and modulated white light data in (C) comprises 12 to 18 infiltrated spots per light treatment. Statistics are Tukey’s multiple comparisons test, ** *P* < 0.05 and *** *P* < 0.01 and **** *P* < 0.001. Leaves were spot infiltrated with OD600 nm = 0.4 *A*. *tumefaciens* cultures. The underlying data for panels C and D is in S6 Data. LED, light-emitting diode.

To deploy the Highlighter system for controlling a chromogenic, readily quantifiable visual reporter, we developed a system variant for biosynthesis of the red pigment betalain. This was achieved by placing the tricistronic RUBY reporter system [44] under Highlighter control, creating Highlighter(RUBY). RUBY is encoded by 3 co-transcribed enzymes, interposed by self-cleaving 2A ribosomal skipping sequences, that catalyze the production of the red pigment betalain from the amino acid tyrosine [44]. Betalain accumulation and reddening of plant tissue provides a visual output which can be readily quantified as a red versus green CIELAB color space component called a*, for which negative values indicate green color and positive values indicate red color. To make Highlighter compatible with RUBY and other plant synthetic biology tools, we ported the system to the MoClo Golden Gate standard [45]. This Golden Gate version of Highlighter(RUBY) consists of 3 transcriptional modules rather than 2, i.e., P_UBQ10_:: *ccaS*_*HL-GG*_::T_HSP_; P_UBQ10_::*ccaR*_*HL-GG*_::T_HSP_; and P_HL_::*RUBY*::T_NOS_, where *ccaS*_*HL-GG*_ and *ccaR*_*HL-GG*_ are Golden Gate compatible variants of *ccaS*_*HL*_ and *ccaR*_*HL*_. A positive control for constitutive RUBY expression was also generated where a 35S promoter replaces P_HL_. As previously, we tested the systems in transiently transformed *N*. *benthamiana* leaves and analyzed RUBY expression (Figs 7 and S7). Under monochromatic light, Highlighter(RUBY) (and a Highlighter(RUBY) variant with nlsTagRFP co-transcribed via a P2A ribosomal skipping sequence with CcaR_HL-GG_), exhibited significantly higher activity in green and red light, when compared to activity levels under blue light (Figs 7B, 7C, and S7). An intermediary activity state with high variability was observed in white light-treated samples. Similarly, under modulated white light conditions, Highlighter(RUBY) showed significantly lower activity in white light modulated with blue light than in white light modulated with red or green light (Fig 7D). The Highlighter(RUBY) results are consistent with Highlighter(YFP) system activity, having somewhat leaky expression in blue light and maximal expression in red light being less than the constitutive control (Figs 7 and S7). The results also mirror our results for Highlighter(NRC4^D478V^) control over HR-associated fluorescence and show that RUBY can be used as a readily quantifiable reporter of system activity, which will permit more rapid engineering of future Highlighter variants.

## Discussion

The development of Highlighter, a cyanobacteriochrome-based light-inducible gene expression system for plants, represents an important step for high-resolution, potentially minimally invasive, and low-cost perturbation of plant biological processes. Advances in plant optogenetics have long been restricted by the limited availability of photoreceptors that are not native to plants and that function independently of exogenously supplied chromophores. Our conversion of the cyanobacterial CcaS-CcaR system for optogenetic control of target gene expression in plants is therefore an important innovation. The Highlighter technology exemplifies how spectrally diverse cyanobacteriochrome-based systems can be repurposed for optogenetic regulation of biological processes in plants, opening up a spectrum of new possibilities.

Ideally, optogenetic actuators in plants should for most applications not photoswitch in standard horticultural light environments where cycling between white light and dark periods is required for plant growth. The complexity of light spectra and light-dark cycling inherent to most growth environments was, therefore, until recently a fundamental challenge to contend with for optogenetic systems in plants. However, the PULSE system [16] elegantly demonstrates that this complication can be circumvented by combining 2 gene-expression switches with competing properties. PULSE combines an SRDX-EL222 “blue-off” module to keep background gene expression low during the light cycle, and a PhyB-PIF “red-on” module, which is activated by monochromatic red light. The Highlighter system, however, has an inherent “blue-repressed” response, without the need for an additional co-expressed module and thus makes it a simpler system for deployment. The unexpected blue light response of the Highlighter system, however, warrants a follow-up investigation to determine its molecular basis in an inherently green/red sensor such as CcaS. Though unforeseen, inactivity in response to blue light is not unprecedented for a CcaS protein. The CcaS homolog from *Nostoc punctiforme* also demonstrated blue-off behavior when repurposed as an optogenetic actuator in *E*. *coli* [46] and for CcaS_HL_, our studies suggest that this response potentially arises from the blue-light-mediated activity of a second CcaS-associated pigment, a flavin.

Although the blue light response of CcaS_HL_ with PΦB in *E*. *coli* was recapitulated *in planta*, several aspects of Highlighter’s response to light stimuli in *N*. *benthamiana* leaves were unexpected. First, system activation in continuous darkness suggests that, when expressed in the absence of a light stimuli, CcaS_HL_ might be biased towards activation. Continuous darkness is, however, a stress condition for *N*. *benthamiana* leaves and thus it is unclear if results in continuous darkness are functionally related to day-night cycling. Indeed, an 8 h dark period within a blue light time series was not sufficient to activate target gene expression. Second, YFP (nlsedAFPt9) transcript levels for Highlighter(YFP) increased and decreased over extended periods in response to the applied light treatments. The former is possibly a result of relatively low transcription activation efficiency, but could also be affected by the presence of an irreversibly blue light inactivated CcaS_HL_ pool. The latter Highlighter inactivation rate could be slowed by a relatively stable phosphorylated CcaR_HL_ and high transcript stability. Third, activation in red light (λ ~ 660 nm) *in planta* stands in contrast to results in *E*. *coli*, again possibly resulting from CcaS_HL_ being biased towards activation in plants. Several non-mutually exclusive mechanisms could explain this unexpected behavior in *N*. *benthamiana* leaves, including differences in temperature (22°C versus 37°C) and cellular environment, an impairment or alteration of GAF domain-mediated light switching (e.g., due to differences in PΦB association efficiency or availability), spectral differences in photosynthetic tissues (e.g., chlorophyll fluorescence under green illumination), or interaction with endogenous signaling components.

Unlike optogenetic systems based on plant photoreceptors, Highlighter is cyanobacterial in origin. This inherent orthogonality theoretically reduces the risk of Highlighter causing undesired off-target phenotypic effects and equally of endogenous light signaling pathways interfering with Highlighter activity. The very low background expression of Highlighter target genes in *N*. *benthamiana* leaves transiently transformed with the ΔCcaS_HL_ and ΔCcaR_HL_ control constructs clearly indicate that there is little endogenous activation of P_HL_ via CcaR_HL_ in the absence of the histidine kinase activity of CcaS_HL_, and similarly when CcaR_HL_, the response regulator, is absent. However, it remains possible that Highlighter, as a two-component system, could still interact to some degree with endogenous plant two-component system signaling components, and it might prove valuable to further orthogonalize the system. Overall, the Highlighter system provides valuable proof-of-principle for converting prokaryotic cyanobacteriochrome-based optogenetic tools for use in eukaryotic plant hosts. The combination of having a GAF domain capable of associating with PΦB and a LOV-like domain associating with a flavin makes Highlighter a potentially versatile chassis for engineering diverse light responses from a single optogenetic tool. Target gene expression control in *N*. *benthamiana* leaves is in the present system best achieved with continuous red and blue light stimuli, while also being compatible to some degree with light-dark cycling and mixed white light environments modulated with blue and red light. Further optimization of the present Highlighter system, e.g., through GAF and LOV-like domain reengineering or CcaR_HL_ and P_HL_ optimization, is needed to limit leaky target gene expression in blue light and maximize target gene induction in other light conditions.

In the future, it will be interesting to develop and implement the suggested system improvements and deploy them in stable transgenic lines expressing Highlighter. To facilitate this process, we have successfully ported Highlighter into a Golden Gate compatible vector system and validated RUBY as a practical reporter of system activity. Given higher throughput in cloning and faster, more direct quantification of system activity levels, we consider Highlighter(RUBY) the system of choice to develop future versions of Highlighter.

With advances in high-resolution quantitation, new hypotheses arise that can only be addressed by perturbing the measured biological process in precisely defined spatial regions and temporal windows. Such studies are often not feasibly conducted using chemically inducible systems because inducer molecules cannot be applied with sufficient resolution. Although future work will address these goals for Highlighter in stable transgenics, our transient expression studies, asserting optogenetic control over fluorescent reporter proteins and plant immunity, demonstrate that Highlighter is already a useful technology that allows precise optogenetic control of target gene expression down to the cellular level and can be deployed to modulate biological processes—even in complex light environments. From our experience using FRET biosensors to investigate how cellular hormone dynamics serve as signal integrators and major regulators of physiology and development [39,47–51], we also recognized a need to precisely perturb cellular hormone dynamics. The development of Highlighter was thus initiated because we envisioned deploying the technology to evaluate hypotheses stemming from high-resolution measurements, for example, distinguishing correlation from causation when investigating the connection between cellular gene expression or metabolite levels, and physiology and development. Beyond the scope of studying endogenous processes, the Highlighter technology holds great potential for plant biotechnology. Highlighter could address bottlenecks in transient *N*. *benthamiana*-based expression platforms for synthesis of high-value compounds and be used to optimally time developmental transitions or stress responses, such as immune activation to ward off pathogen outbreaks in greenhouse or vertically farmed crops. We therefore expect Highlighter to become a resource in the plant optogenetic toolbox, complementing PULSE and other exciting recent developments in the field [12,16,52–54], and changing how we approach hypothesis testing in plant biology and how we address production and yield bottlenecks in plant biotechnology.

## Materials and methods

A detailed description of the plasmids used in this article, and their assembly, is found in S1 Table. PCR primers were synthesized by Sigma Aldrich (S2 Table), longer DNA fragments and genes were ordered from GeneScript (S3 Table). PCRs were performed using Q5 High-Fidelity DNA Polymerase (New England Biolabs (NEB), Cat#M0491S/L) and gel extractions were done with the Macherey-Nagel NucleoSpin Gel and PCR Clean-up Mini Kit (Macherey-Nagel, Cat#740609). DNA assemblies were carried out by In-Fusion Cloning (Takara Bio, In-Fusion HD Cloning Plus kit, Cat#638909) or NEBuilder assembly (NEB, NEBuilder High-Fidelity Master Mix, Cat#M5520) as per manufacturer’s instructions. Assembly reactions were transformed into chemically competent *E*. *coli* cells: Stellar competent cells (Takara Bio, Cat#636763), chemically competent DH5α cells or NEB 10-beta competent cells (NEB, Cat#C3019). Constructs were selected on LB plates (1% Tryptone, 0.5% Yeast Extract, and 1% Sodium Chloride 1.5% Bacto agar) with appropriate selection. Plasmid purification was performed using the Qiagen QIAprep Spin Miniprep Kit (Qiagen, Cat#27106). Plasmids were verified by restriction enzyme digestion and sequencing (Sanger sequencing, Source BioScience). Site directed mutagenesis was performed using primers designed using the QuikChange Primer Design tool by Agilent Technologies with QuikChange II Kit settings (https://www.agilent.com/store/primerDesignProgram.jsp).

*E*. *coli* strains were prepared for bacterial photoswitching experiments by co-transforming *E*. *coli* DH5α cells with vector sets for expressing the CcaS-CcaR system and system variants. One vector (based on pSR43.6r) expressed CcaS, or a CcaS variant, and genes for either PCB or PΦB biosynthesis, and a second vector (pBL413-003-020, derived from pSR58.6) expressed CcaR and further encoded an sfGFP reporter cassette where *sfgfp* is under the control of the engineered cognate promoter for CcaR, P_cpcG2-172_ [20]. Liquid *E*. *coli* cultures expressing CcaS-CcaR system variants were cultured in darkness for 12 to 14 h in LB (1% Tryptone, 0.5% Yeast Extract, 1% Sodium Chloride) with appropriate antibiotics in 96-well plates (VWR, Cat#732–3802), with one 3 mm glass bead and 750 μl media per well at 37°C, shaking at 220 rpm. Cultures were serial diluted in LB from 3-fold to 2,187-fold in 96-well plates (Thermo Fisher Scientific, Greiner Bio-One Cat#655101) and incubated at 37°C, shaking (250 rpm) while receiving light treatments. Light treatments were approximately 10 μmol m^-2^ s^-1^ light from LEDs with peak emissions around 400 nm, 455 nm, 525 nm, 590 nm, 605 nm, 630 nm, 660 nm, and 695 nm. Complete spectra and LED models are found in S8 Fig. Light intensities were measured using a Licor LI-250A light meter with the LI-190R Quantum Sensor and spectra were recorded using an UPRtek MK350S LED meter. sfGFP fluorescence was quantified on a fluorimeter (Molecular devices, SpectraMax i3x - fluorescence read with 5 points, 6 flashes per well, bottom read, excitation 485 nm ± 4.5 nm, and emission 516 nm ± 7.5 nm), along with the cell density (absorbance read at 600 nm, endpoint). For quantification of induction, fluorescence counts (LB fluorescence background subtracted) were plotted against cell densities (LB absorbance background subtracted). Fluorescence at OD600 nm = 0.2 was estimated from the plots with third order polynomial trendlines.

For heterologous expression, purification, and spectroscopy of holo-CcaS_HL_, *E*. *coli* expressing hexahistidine tagged CcaS_HL_ alongside PΦB biosynthetic enzymes HO1 and mHY2 were cultured in 24 L of LB medium at 18°C. The purification protocol consisted of 3 steps performed on an ÄKTA Pure System (Cytiva): immobilized metal-affinity chromatography (Cytiva, 5 mL HisTrap HP, Cat#17524802) for hexahistidine tagged CcaS_HL_, ion-exchange chromatography with a linear gradient of salt (Cytiva, 5 mL HiTrap Q HP, Cat#17115401) and size-exclusion chromatography (SEC) (Cytiva, HiLoad 26/600 Superdex 200 pg, Cat#28989336). This expression and purification protocol yielded 3 mL of the target at approximately 18 μm (determined from 280 nm absorbance of “post-purification” sample). Absorbance spectra (300 to 800 nm) for the resulting product was analyzed on a spectrometer (Agilent, Cary 60) following purification and then following illumination with light from the ColorDyne Benchtop Lightsource at the wavelengths and periods of time indicated in S2 Fig. In any given figure panel in S2 Fig, the same sample was illuminated for cumulative periods followed by data acquisition (e.g., illumination for 1 min, followed by data acquisition (spectrum “1 min”); illumination for a further minute, followed by data acquisition (“2 min”); illumination for a further 3 min, followed by data acquisition (“5 min”)). Fluorescence emission spectra were acquired between 460 and 800 nm (S3 Fig) following photoexcitation at 445 nm of the red-absorbing state of CcaS_HL_ (to avoid further photoisomerisation of the PΦB chromophore during measurement).

*Agrobacterium*-mediated transient transformation and photoswitching assays in *N*. *benthamiana* were performed by transforming electrocompetent *A*. *tumefaciens* GV3101, carrying the pMP90 helper plasmid [55], with Highlighter plasmids in 1 mm electroporation cuvettes (Eurogentec, Cat#CE-0001-50) using an Eppendorf Multiporator (Cat#4308, 1500 V τ 5 ms). Cells recovered for 1 to 2 h in LB at 28°C and were selected on LB plates supplemented with appropriate antibiotics. *A*. *tumefaciens* strains carrying plasmids for testing Highlighter system variants *in planta* were cultured at 28°C in liquid LB media, shaking at 220 rpm, supplemented with appropriate antibiotics. Cultures were pelleted, washed, and resuspended in infiltration media (10 mM MES, 10 mM MgCl_2_, 200 μm Acetosyringone (Sigma Aldrich, Cat#D134406), pH 5.6) to an OD600 nm of 0.2 to 0.4 and mixed equally with *A*. *tumefaciens* C58C1 cells carrying the p19 plasmid, encoding the p19 RNA-silencing suppressor from *Tomato bushy stunt virus* [56]. Four-week-old leaves were syringe infiltrated through the abaxial side and left in the dark for 8 to 16 h before undergoing light treatments. For light treatments, infiltrated leaves were cut from plants and placed on 1% water agarose plates, abaxial side up, and sealed with surgical tape. Light treatments of infiltrated leaves were performed using Heliospectra lamps (model RX30) with total light intensities of 100 μmol m^-2^ s^-1^. Monochromatic LED light regimes were generated using the 450 nm blue light channel (λ ~ 455 nm), 530 nm green light channel (λ ~ 525 nm), 620 nm orange light channel (λ ~ 630 nm), and 660 nm red light channel (λ ~ 660 nm). Modulated white light regimes, also referred to as mixed or enriched white light regimes, were defined as 50 μmol m^-2^ s^-1^ light from 5700 K white light LEDs, enriched with 50 μmol m^-2^ s^-1^ light from one of the abovementioned blue, green, and red LED channels. Light intensities were measured using a Licor LI-250A light meter with a LI-190R Quantum Sensor and spectra were recorded using an UPRtek MK350S LED meter (S10 Fig).

HR responses were scored 4 to 5 days after infiltration via accumulation of HR-associated fluorescent compounds in infiltrated spots using a Syngene G-BOX (Model F3-LFP, UV Transilluminator; manual capture mode, TLUM lighting, UV032 filter). UV-fluorescence response resulting from Highlighter induced NRC4^D478V^ expression was defined as follows:

$$HR\;fluorescence=\frac{Inducible\;construct\;fluorescence-Negative\;control\;fluorescence}{Positive\;control\;fluorescence-Negative\;control\;fluorescence}.$$

Fluorescent signals from nlsedAFPt9 and nlsTagRFP were collected by confocal imaging using a Leica TSC SP8 laser scanning confocal microscope. nlsedAFPt9 and nlsTagRFP were simultaneously excited with a 514 nm Argon laser; YFP emission was collected from 520 to 540 nm and RFP emission was collected from 595 to 625 nm on HyD detectors. Segmentation and quantification of fluorescence intensities were performed in ImageJ. 3D segmentation was performed using the 595 to 625 nm RFP channel and induction ratios were calculated as nuclear YFP signals divided by nuclear RFP signals. Overexposed voxels were excluded from the analysis when relevant.

For high-resolution laser illumination to control target gene (nlsedAFPt9) expression levels, infiltrated plants were kept in darkness for 12 to 16 h post infiltration and continuously treated with blue light (100 μmol m^-2^ s^-1^ light λ ~ 455 nm) until 2.5 days post infiltration. Infiltrated leaves were then cut off plants and transferred to 1% water agarose plates and placed under the objective on the confocal microscope. Cling film was used to seal the space between the plate and objective to maintain adequate humidity for sample health. A region with cells with early detectable nuclear localized RFP fluorescence was selected for time lapse imaging of nlsedAFPt9 expression. Light treatments were performed with a 442 nm laser (40 mW, 442 nm Diode laser at 0.45%) and a 633 nm laser (10 mW 633 nm HeNe laser at 0.15%) on a Leica TSC SP8 microscope using the FRAP module. Samples were light treated for 7 h and imaged. Light treatment and imaging cycles were repeated up to 5 times.

Quantification of betalain production, i.e., quantification of RUBY reporter activity via the redness of infiltrated spots, was performed 3 to 5 days post infiltration. To quantify redness, leaves were first imaged using a high-resolution flatbed photo scanner and secondly quantified using a method developed by Vivian Zhong and Ian Kinstlinger over Twitter [57]. Specifically, images were converted from RGB (Red, Green, and Blue) color space to CIELAB at the D65 white point using the Color Space Converter ImageJ plugin and the red-green component a* was measured using ImageJ. Subsequent data processing and graphical data visualization was performed using custom Python code, integrating the Pandas and Seaborn libraries [58,59].

For qRT-PCR quantification of gene expression levels, RNA was isolated from infiltrated, light-treated *N*. *benthamiana* leaf discs frozen in liquid nitrogen. Total RNA was extracted using the RNeasy Plant Mini Kit (Qiagen, Cat#74904) and DNase treated with the Invitrogen TURBO DNA-free Kit (Thermo Fisher Scientific, Cat#AM1907). cDNA was synthesized with the SuperScript VILO cDNA Synthesis Kit (Thermo Fisher Scientific, Cat#11754–250). Gene expression levels in samples were determined in quadruplicate by qPCR using gene-specific primers (5′GAAGAGAAAGGTTGGAGGGCT3′ and 5′TGACCGAAAACTTATGCCCGT3′ for nlsedAFPt9; 5′TGTGTCAGGGAAAGAATGGAG3′ and 5′TCAGAACCGAGCATATCGAG3′ for CcaR_HL_), a Lightcycler 480 (Roche Molecular Systems, Cat#05015243001) and qPCR LightCycler 480 SYBR Green I Master (Roche Molecular Systems Cat# 04887352001) according to manufacturer’s instructions. Target gene (nlsedAFPt9) expression levels were quantified using the delta-delta Ct method [60], using CcaR_HL_ as the calibrator gene.

Differential target gene expression in response to light treatments was confirmed by one-way ANOVA, equality of group variances validated by Brown–Forsythe test, and multiplicity adjusted *P*-values from Tukey’s multiple comparison test were depicted on graphs.

## Supporting information

S1 Fig Alignment of GAF domains from cyanobacteriochromes, plant phytochromes and bacterial phytochromes. (DOCX)

S2 Fig Spectroscopic characterization of holo-CcaS_HL_ purified from PΦB-producing *E*. *coli*. (DOCX)

S3 Fig Fluorescence emission spectrum for holo-CcaS_HL_ purified from PΦB-producing *E*. *coli*. (DOCX)

S4 Fig Highlighter(YFP) expression control behavior in *N*. *benthamiana* leaves in response to monochromatic light treatments and darkness. (DOCX)

S5 Fig Absolute target gene expression levels in *N*. *benthamiana* leaves in response to monochromatic blue and red light stimuli. (DOCX)

S6 Fig Cellular resolution measurements of nuclear YFP/RFP ratios, from Fig 5C, generated by deploying Highlighter(YFP) in transiently transformed *N*. *benthamiana*. (DOCX)

S7 Fig Comparison of Highlighter controlled expression of betalain-producing RUBY reporter in monochromatic light conditions. (DOCX)

S8 Fig Light spectra of LED arrays used for light treating *E*. *coli* cultures expressing the CcaS&CcaR system variants. (DOCX)

S9 Fig Light spectra of LEDs used for the spectroscopic characterization of holo-CcaS_HL_ in S2 Fig. (DOCX)

S10 Fig Light spectra for Heliospectra RX30 lamps. (DOCX)

S1 Table Vectors insert and construction description. (DOCX)

S2 Table Primers used to assemble vectors in S1 Table. (DOCX)

S3 Table Synthesized genes used as PCR templates for vector assemblies. (DOCX)

S1 Data Data underlying Fig 2. (XLSX)

S2 Data Data underlying Fig 3B and 3C. (XLSX)

S3 Data Data underlying Fig 4. (XLSX)

S4 Data Data underlying Figs 5C, 5D, and S6. (XLSX)

S5 Data Data underlying Fig 6D and 6E. (XLSX)

S6 Data Data underlying Figs 7C, 7D, and S7. (XLSX)

S7 Data Data underlying S1C Fig. (XLSX)

S8 Data Data underlying S4 Fig. (XLSX)

S9 Data Data underlying S5A and S5B Fig. (XLSX)

## Abbreviations

CRE: *cis*-regulatory element
FMN: flavin mononucleotide
FRAP: Fluorescence Recovery After Photobleaching
HR: hypersensitive response
IR-LEGO: infrared laser-evoked gene operator
LED: light-emitting diode
NLS: nuclear localization signal
PCB: phycocyanobilin
SEC: size-exclusion chromatography
sfGFP: superfolder green fluorescent protein
TAD: trans-activation domain
TMD: transmembrane domain
YFP: yellow fluorescent protein
