# Supplementary material for: Highlighter: An optogenetic system for high-resolution gene expression control in plants
Source: PLoS Biol. 2023 Sep 21;21(9):e3002303. doi: 10.1371/journal.pbio.3002303 (PMC10513317; doi:10.1371/journal.pbio.3002303)

**S1 Fig. Alignment of GAF domains from cyanobacteriochromes, plant phytochromes and bacterial phytochromes.** (A) GAF domains from selected cyanobacteriochromes, plant phytochromes and bacterial phytochromes were aligned to identify functionally conserved amino acid residues involved in chromophore accommodation. In this alignment, the native chromophore of each GAF domain is indicated in parenthesis. The alignment below specifically shows the part of the alignment from the conserved DRV motif to the conserved LWG motif. Residues in the CcaS sequence from *Synechocystis* sp. PCC6803 (SyCcaS), marked in green, were individually mutated to corresponding amino acids marked in yellow to identify modifications that could improve CcaS photoswitching with PΦB. Details for the cyanobacteriochromes, plant phytochromes and bacterial phytochromes included in the alignment are presented in the table below the alignment (B). The alignment was performed in CLC Main Workbench 7.6.4 with the following settings: Gap open cost = 10.0, Gap extension cost = 1.0, End gap cost = As any other, Alignment mode = Very accurate, Redo alignments = No, Use fixpoints = No. BV = Biliverdin, PCB = Phycocyanobilin, PVB = Phycoviolobilin and PΦB = Phytochromobilin. (C) The eight mutations were individually introduced into CcaS (constructs pBL413-006-056 to pBL413-006-063) and screened for their ability to induce sfGFP fluorescence in response to green light stimuli in *E. coli* with PΦB as the chromophore. System induction in response to green light stimuli was quantified as relative fluorescence arising from sfGFP fluorescence after green light treatment divided by sfGFP fluorescence after dark treatment. Green light stimuli (~10 µmol m^-2^ s^-1^), were generated using LEDs with peak wavelength emission around 530 nm. CcaS(A92V) with PΦB and CcaS with its native PCB chromophore responded similarly to the green light treatment. The underlying data for panel C is in S7 Data.


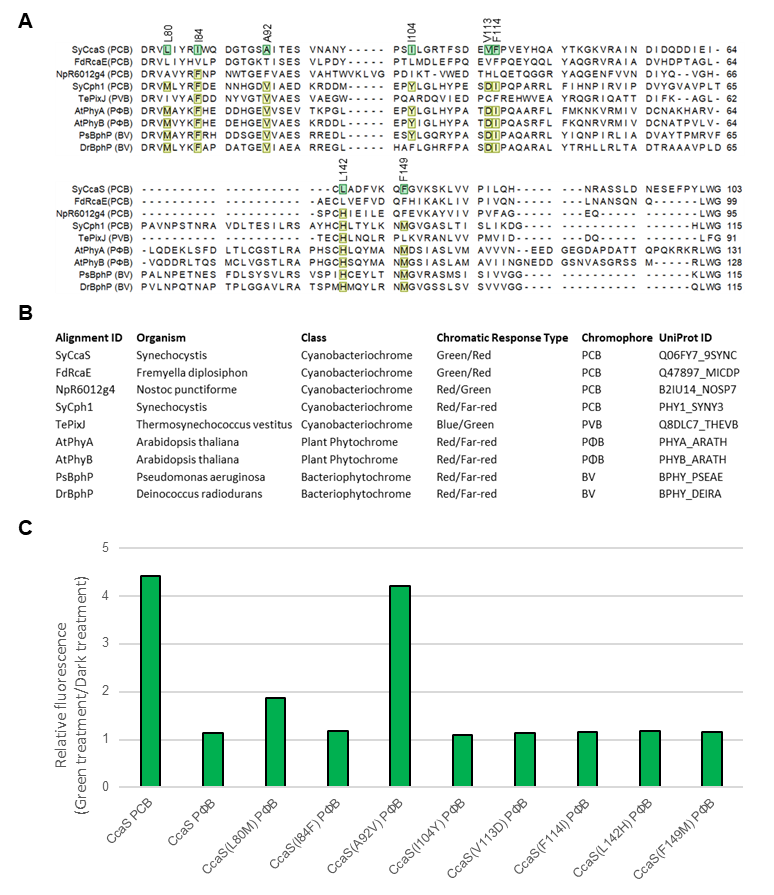

Supplement: S1 Fig — (DOCX) [file pbio.3002303.s001.docx]
