# Supplementary material for: Highlighter: An optogenetic system for high-resolution gene expression control in plants
Source: PLoS Biol. 2023 Sep 21;21(9):e3002303. doi: 10.1371/journal.pbio.3002303 (PMC10513317; doi:10.1371/journal.pbio.3002303)

**S4 Fig. Highlighter(YFP) expression control behavior in *N. benthamiana* leaves in response to monochromatic light treatments and darkness**. Relationship between light treatments and resulting target gene expression control by Highlighter. Highlighter(YFP) expression, as presented in Figure 3B, compared with negative controls (ΔCcaS_HL_ & ΔCcaR_HL_) and a positive control (constitutive YFP) (Vector IDs pBL413-024-257, pBL413-024-260, pBL413-024-261 and pBL413-024-259, respectively, (S1 Table)). *N. benthamiana* leaves were infiltrated with *A. tumefaciens* for delivery of Highlighter(YFP) and corresponding control constructs. Infiltrated leaves were kept dark overnight before receiving treatments for 3 days with continuous blue, green and red light or darkness. Light was delivered with LEDs (100 µmol m^-2^ s^-1^) with peak wavelength emissions λ ~ 455 nm, 525 nm and 660 nm, respectively. Means and S.E.M. are presented for 3 biological independent experiments for each evaluated construct. Individual means are depicted with circles colored according to light treatment. n per biological mean in is 22 to 259 nuclei from 3 to 4 infiltrated spots across 3 to 4 leaves. * P<0.05 and ** P<0.01. Leaves were spot infiltrated with OD600 nm = 0.4 *A. tumefaciens* cultures. The underlying data is in S8 Data.


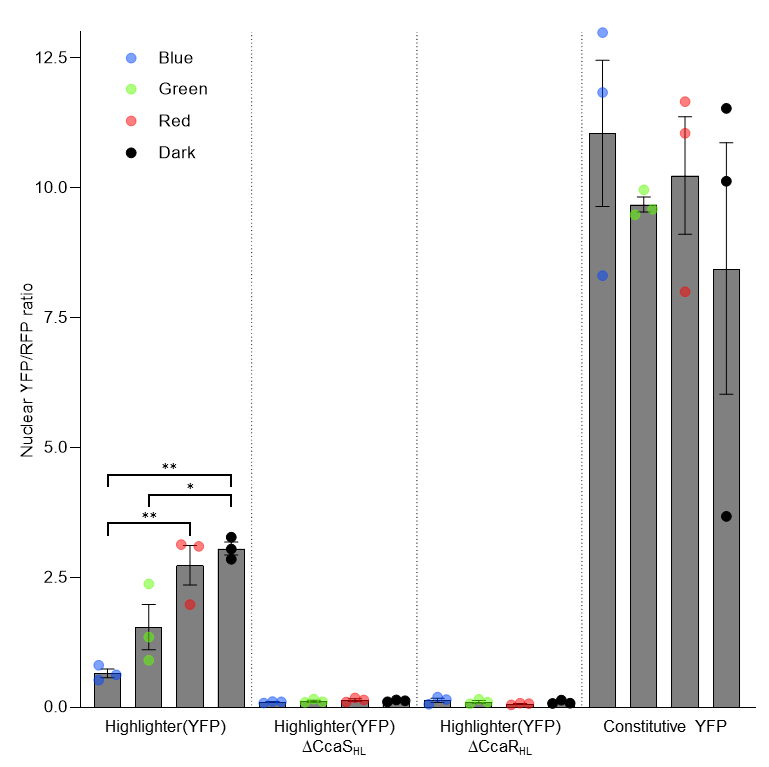

Supplement: S4 Fig — (DOCX) [file pbio.3002303.s004.docx]
