# Supplementary material for: Highlighter: An optogenetic system for high-resolution gene expression control in plants
Source: PLoS Biol. 2023 Sep 21;21(9):e3002303. doi: 10.1371/journal.pbio.3002303 (PMC10513317; doi:10.1371/journal.pbio.3002303)

**S5 Fig. Absolute target gene expression levels in *N. benthamiana* leaves in response to monochromatic blue and red light stimuli.** Absolute target gene transcript levels generated by the Highlighter system in transiently transformed *N. benthamiana* in response to blue and red light treatments. (A) Non-normalized YFP transcript levels relating to Figure 5D, graphed as 2^-Ct^. (B) Non-normalized YFP transcript levels for the Highlighter(YFP) ΔCcaS_HL_ negative control, graphed as 2^-Ct^. As described for Figure 5, *N. benthamiana* leaves infiltrated with Agrobacterium for delivery of Highlighter(YFP) (A) or Highlighter(YFP) ΔCcaS_HL_ (B), were kept in darkness overnight and treated continuously with blue light (100 µmol m^-2^ s^-1^, λ ~ 455) until 2.5 days post infiltration (here defined as 0 h) to minimize target gene expression levels. Infiltrated leaves were then exposed to blue or red LED light treatments (λ ~ 455 nm and 660 nm, respectively, 100 µmol m^-2^ s^-1^) for 36 h and leaf tissue was sampled every 12 h. Means are presented as spots (colored according to light treatment post 0 h) for each time point for 3 biological independent experiments. For each time point in each of the biological replicates, i.e. for each spot, leaf material from 4 infiltrated spots across 4 leaves were polled for analysis using quadruple technical qPCR replicates. (C) & (D) Nuclear YFP fluorescence in laser illuminated *N. benthamiana* samples expressing Highlighter(YFP) (C) and the constitutive YFP expression control (D), relating to presented data in Fig 5A and 5B. As described, samples were blue light treated until 2.5 days post infiltration to minimize target gene expression and then subjected to blue and red light treatments with lasers (initiation of laser treatments are defined as 0 h). 442 nm blue and 633 nm red lasers were used to irradiate the area outlined in blue and red. Highlighter(YFP) Vector ID: pBL413-024-257; Highlighter(YFP) ΔCcaS_HL_ Vector ID: pBL413-024-260; Constitutive YFP control Vector ID: pBL413-024-259 (S1 Table). Leaves were spot infiltrated with OD600 nm = 0.4 *A. tumefaciens* cultures. The underlying data for panels A and B is in S9 Data.


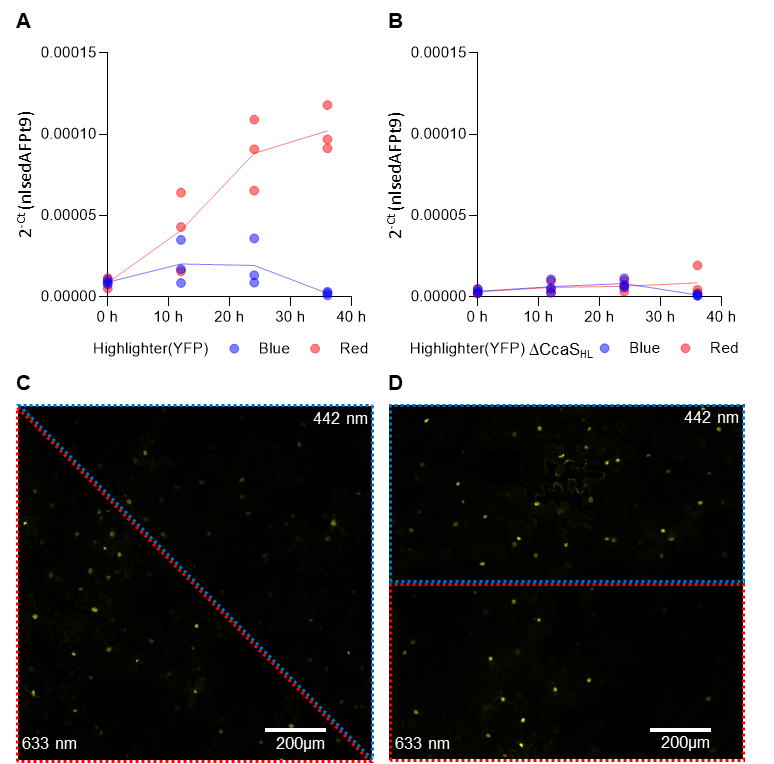

Supplement: S5 Fig — (DOCX) [file pbio.3002303.s005.docx]
