# Supplementary material for: Highlighter: An optogenetic system for high-resolution gene expression control in plants
Source: PLoS Biol. 2023 Sep 21;21(9):e3002303. doi: 10.1371/journal.pbio.3002303 (PMC10513317; doi:10.1371/journal.pbio.3002303)

**S7 Fig. Comparison of Highlighter controlled expression of betalain-producing RUBY reporter in monochromatic light conditions.** N. benthamiana leaves were infiltrated with the betalain-producing Highlighter(RUBY) reporter construct in which CcaR_HL-GG_ was co-transcribed with a nlsTagRFP reporter via a P2A ribosomal skipping sequence (Vector ID pRH-19-781 (S1 Table)) and a corresponding negative control lacking CcaS_HL-GG_ expression (ΔCcaS_HL-GG_, Vector ID pRH-19-783 (S1 Table)). Plants were moved from darkness to 100 μmol m^-2^ s^-1^ monochromatic or white light treatments after 12 hours (100W - white (5700K), 100B - blue light (λ ~ 455 nm), 100G - green light (λ ~ 525 nm), 100R - red light (λ ~ 660 nm). RGB leaf images acquired after 3-5 days were processed into the CIELAB color space and mean a* values were quantified for uninfiltrated and vector infiltrated spots. Each data point represents an individual spot. The experiment was completed twice with similar results. The underlying data is in S6 Data.


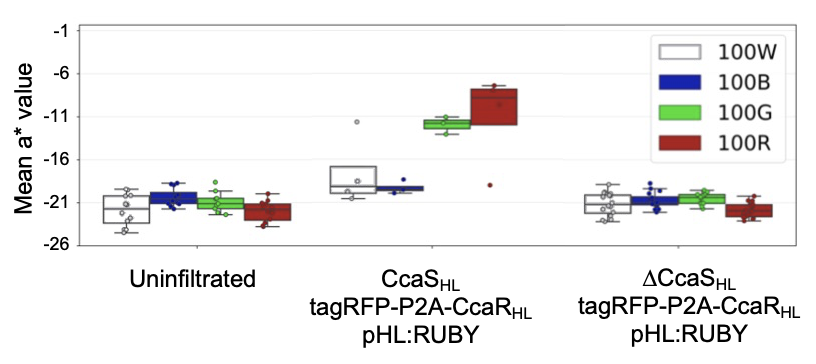

Supplement: S7 Fig — (DOCX) [file pbio.3002303.s007.docx]
