# Supplementary material for: Highlighter: An optogenetic system for high-resolution gene expression control in plants
Source: PLoS Biol. 2023 Sep 21;21(9):e3002303. doi: 10.1371/journal.pbio.3002303 (PMC10513317; doi:10.1371/journal.pbio.3002303)
Supplement: S9 Fig — (DOCX) [file pbio.3002303.s009.docx]

**S9 Fig**. **Light spectra of LEDs used for the spectroscopic characterization of holo-CcaS_HL_ in S2 Fig.**
