# Supplementary material for: Highlighter: An optogenetic system for high-resolution gene expression control in plants
Source: PLoS Biol. 2023 Sep 21;21(9):e3002303. doi: 10.1371/journal.pbio.3002303 (PMC10513317; doi:10.1371/journal.pbio.3002303)

**S10 Fig**. **Light spectra for Heliospectra RX30 lamps**. Light spectra for light regimes generated with Heliospectra RX30 lamps (Company information in M&M) for light infiltrated *N. benthamiana* leaves. Light spectra were recorded with an UPRtek MK350S LED meter. **a**. 450 nm LED channel, **b**. 530 nm LED channel, **c**. 620 nm LED channel, **d**. 660 nm LED channel **e**. 5700 K LED channel (white light LED channel), **f**. Blue enriched white light; 1:1 ratio of 450 nm LED channel and 5700 K LED channel, **g**. Green enriched white light; 1:1 ratio of 530 nm LED channel and 5700 K LED channel **h**. Orange enriched white light; 1:1 ratio of 660 nm LED channel and 5700 K LED channel.


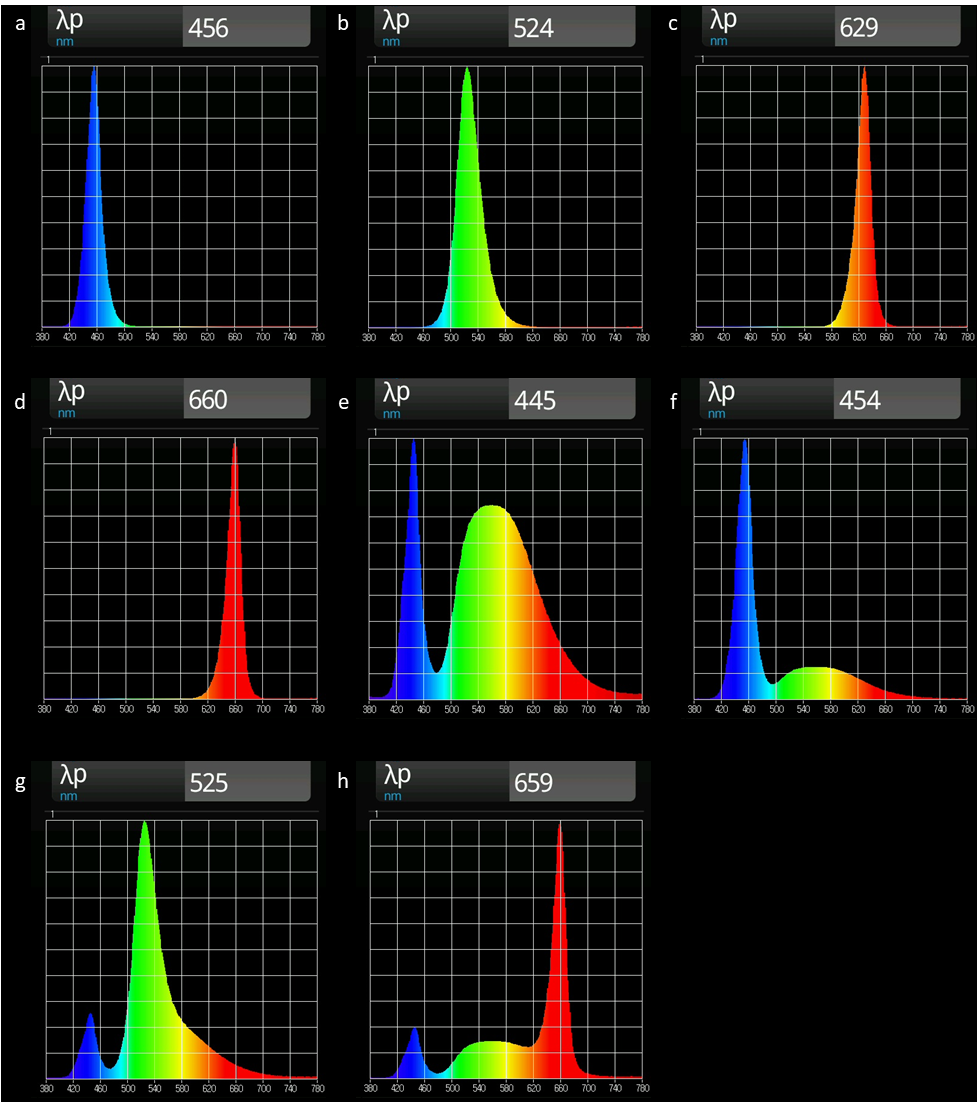

Supplement: S10 Fig — (DOCX) [file pbio.3002303.s010.docx]
