# Supplementary material for: Highlighter: An optogenetic system for high-resolution gene expression control in plants
Source: PLoS Biol. 2023 Sep 21;21(9):e3002303. doi: 10.1371/journal.pbio.3002303 (PMC10513317; doi:10.1371/journal.pbio.3002303)
Supplement: S3 Table — (DOCX) [file pbio.3002303.s013.docx]

| **S3 Table.** Synthesized genes used as PCR templates for vector assemblies. |
| --- |
| Gene: nlsCcaS(A92V del1_87) codon-optimized for *Arabidopsis*  Legend: Start codons NLS CcaS(A92V del1_87)  Sequence:ATGATGTTACAACCAAAGAAGAAAAGGAAGGTGGGTGGAAGAAGAATAGAAATAAGTATCAAGCAGCAGACACAACGTGAGAGGTTTATCAACCAAATCACACAGCATATCAGACAATCTCTTAATTTGGAGACTGTTTTGAACACTACAGTTGCTGAAGTTAAGACACTTTTGCAGGTTGATAGAGTTCTTATCTATAGAATCTGGCAAGATGGTACAGGATCTGCTATCACTGAGTCTGTTAATGCTAACTACCCTTCTATTTTGGGTAGAACTTTTTCTGATGAGGTTTTCCCAGTTGAATATCATCAAGCTTACACAAAGGGAAAAGTTAGAGCTATTAATGATATCGATCAGGATGATATCGAAATCTGTCTTGCTGATTTCGTTAAACAATTCGGTGTTAAGTCTAAACTTGTTGTTCCTATCTTGCAGCATAATAGAGCTTCTTCTTTGGATAACGAATCTGAGTTTCCATATCTTTGGGGACTTTTGATTACACATCAGTGTGCTTTCACTAGACCTTGGCAACCTTGGGAAGTTGAGCTTATGAAGCAGTTGGCTAACCAAGTTGCTATTGCTATCCAACAGTCTGAGTTGTACGAACAACTTCAACAGTTGAATAAGGATCTTGAGAACAGAGTTGAAAAAAGAACACAACAGTTGGCTGCTACTAATCAGTCTCTTAGGATGGAAATCTCTGAAAGACAAAAGACTGAGGCTGCTTTGAGACATACTAACCATACACTTCAGTCTTTGATTGCTGCTTCTCCTAGAGGTATCTTTACTCTTAATTTGGCTGATCAAATTCAGATCTGGAACCCAACAGCTGAGCGAATCTTCGGATGGACTGAAACAGAGATTATCGCTCATCCTGAGCTTTTGACATCTAACATCCTTTTGGAAGATTACCAACAGTTTAAGCAAAAGGTTCTTTCTGGTATGGTTTCTCCATCTCTTGAGTTGAAGTGTCAGAAGAAAGATGGATCTTGGATTGAAATCGTTTTGTCTGCTGCTCCTCTTTTGGATTCTGAAGAGAACATTGCTGGTCTTGTTGCTGTTGTTGCTGATATCACTGAGCAAAAAAGACAGGCTGAACAAATCAGACTTTTGCAATCTGTTGTTGTTAACACAAACGATGCTGTTGTTATTACTGAAGCTGAACCAATCGATGATCCTGGACCAAGAATCCTTTATGTTAATGAGGCTTTCACTAAGATCACAGGATACACTGCTGAAGAGATGTTGGGAAAGACTCCTAGAGTTCTTCAAGGACCAAAAACTTCAAGAACTGAGTTGGATAGAGTTAGACAGGCTATCTCTCAATGGCAGTCTGTTACAGTTGAAGTTATTAATTACAGAAAGGATGGTTCTGAGTTTTGGGTTGAATTTTCTCTTGTTCCTGTTGCTAACAAAACAGGATTTTACACTCATTGGATTGCTGTTCAAAGAGATGTTACAGAGAGAAGAAGAACTGAAGAGGTTAGACTTGCTTTGGAAAGAGAGAAGGAACTTTCAAGATTGAAGACTAGATTTTTCTCTATGGCTTCTCATGAGTTTAGAACACCACTTTCTACTGCTTTGGCTGCTGCTCAACTTCTTGAAAATTCTGAAGTTGCTTGGCTTGATCCTGATAAGAGATCAAGAAACCTTCATAGAATCCAAAATTCTGTTAAAAACATGGTTCAACTTTTGGATGATATCTTGATTATCAACAGAGCTGAGGCTGGAAAGCTTGAGTTTAATCCAAACTGGCTTGATTTGAAGCTTTTGTTCCAACAGTTCATTGAAGAGATCCAGCTTTCTGTTTCTGATCAATACTACTTCGATTTCATCTGTTCTGCTCAAGATACTAAGGCTCTTGTTGATGAAAGATTGGTTAGATCTATCCTTTCTAATCTTTTGTCTAACGCTATCAAGTACTCTCCTGGAGGTGGACAGATTAAAATCGCTCTTTCTTTGGATTCTGAGCAGATTATCTTCGAAGTTACAGATCAAGGTATTGGAATCTCTCCTGAGGATCAAAAGCAGATCTTTGAACCATTCCATAGAGGAAAGAATGTTAGAAACATTACTGGTACAGGACTTGGTTTGATGGTTGCTAAGAAATGTGTTGATCTTCATTCTGGATCTATCCTTTTGAAGTCTGCTGTGGATCAAGGAACAACTGTGACCATCTGTCTCAAAAGGTACAACCATCTCCCAAGGGCT |
| Gene: nlsCcaR Codon-optimized for *Arabidopsis*  Legend: Start codons NLS CcaR(del1_3) VP16  Sequence:ATGATGTTGCAGCCTAAAAAGAAGAGAAAAGTTGGTGGTAGAATACTCCTCGTGGAAGATGATTTGCCATTAGCAGAAACCCTCGCAGAAGCTTTGTCTGATCAACTTTACACTGTTGATATTGCTACAGATGCTTCTTTGGCTTGGGATTATGCTTCTAGACTTGAATACGATTTGGTTATTCTTGATGTTATGTTGCCTGAGCTTGATGGAATTACTCTTTGTCAGAAGTGGAGATCTCATTCTTATTTGATGCCAATCCTTATGATGACTGCTAGAGATACAATTAATGATAAGATCACAGGACTTGATGCTGGTGCTGATGATTACGTTGTTAAACCTGTTGATTTGGGTGAACTTTTTGCTAGAGTTAGAGCTCTTTTGAGAAGAGGATGTGCTACTTGTCAACCAGTTTTGGAGTGGGGTCCTATTAGACTTGATCCATCTACTTATGAAGTTTCTTACGATAATGAGGTTTTGTCTCTTACAAGAAAGGAATACTCTATCTTGGAGCTTTTGCTTAGAAACGGAAGAAGAGTTCTTTCTAGATCTATGATCATCGATTCTATCTGGAAGTTGGAGTCTCCTCCAGAAGAGGATACAGTTAAAGTTCATGTTAGATCTTTGAGACAAAAGCTTAAGTCTGCTGGACTTTCTGCTGATGCTATTGAAACTGTTCATGGAATCGGTTACAGATTGGCTAATCTTACAGAGAAGTCTTTGTGTCAGGGAAAGAATTCTTCTGCTCCTCCAACTGATGTTTCTCTTGGAGATGAATTGCATCTTGATGGAGAGGATGTTGCTATGGCTCATGCTGATGCTTTGGATGATTTTGATTTGGATATGCTTGGAGATGGAGATTCTCCTGGACCAGGTTTCACACCTCATGATTCTGCTCCATACGGTGCTCTTGATATGGCTGATTTTGAGTTTGAGCAGATGTTCACTGATGCACTTGGTATTGATGAGTATGGAGGA |
